# Encapsulated miR-200c and Nkx2.1 in a nuclear/mitochondria transcriptional regulatory network of non-metastatic and metastatic lung cancer cells

**DOI:** 10.1186/s12885-019-5337-6

**Published:** 2019-02-11

**Authors:** Olga D’Almeida, Omar Mothar, Esther Apraku Bondzie, Yolande Lieumo, Laure Tagne, Sumeet Gupta, Thomas Volkert, Stuart Levine, Jean-Bosco Tagne

**Affiliations:** 1 The Pulmonary Center, Boston University School of Medicine (BUSM), East Concord Street R304, Boston, MA 02118 USA; 2 Faculté de Pharmacie, Université D’Auvergne, Clermont Ferrand, France; 3 Whitehead Institute for Biomedical Research (WIBR), Nine Cambridge Center Cambridge, Cambridge, MA 02142 USA; 4 Department of Biology, Massachusetts Institute of Technology (MIT), Cambridge, MA 02139 USA

**Keywords:** Nano formulation, Zeta (ζ) potential, Encapsulation, Nkx2.1, microRNA-200c, Lung cancer, Metastasis, Kras, EMT, Hippo pathways

## Abstract

**Background:**

MicroRNAs are noncoding RNA molecules of ~ 22 nucleotides with diagnostic and therapeutic action [Curr Drug Targets, 2015. 16(12): p. 1381-403], affecting the expression of mRNAs involved in invasion, migration, and development [Oncotarget, 2015. 6(9): p. 6472-98, Cancer Manag Res, 2014. 6: p. 205-16]. miR-200c is part of the miR-200c/141 cluster on chromosome 12p13. Its mechanism of action when encapsulated is critical in lung cancer when patients express changes in miRNAs. miR-200c be a potential biomarkers for various lung diseases. As a potential therapy, miR-200c can impacts lives as target lung cancer is a leading cause of death with about 234,000 cases annually, high heterogeneity, complex screening, and a 5-year survival rate of 16% [CA Cancer J Clin, 2016.66(1): p. 7-30]. Encapsulated miR-200c efficiently enhances bioavailability, pharmacokinetics of therapeutics and targeting to cells, improves efficacy and provides potential cure.

**Methods:**

The functions of miR-200c were determined in non-metastatic KW-634 and metastatic 821-T4 and 821-LN mouse lung cancer cell lines after various Nano vehicle treatments. Viability and cytotoxicity were determined by cell cycle and quantitative real-time PCR analyses were used to quantify levels of miR-200c and its target genes. In situ hybridization was used to visualize patterns of expression *of miR-200c and others* in the lung and many organs. Next-generation sequencing accession number GSE125000, invasion and migration assays using transwell chambers, and ActivSignal were used to elucidate the activation and inhibition profiles and perform direct expression measurements and modification of cellular components.

**Results:**

Due to their effectiveness as intracellular vesicles transporting miR-200c into, out, and between parts of the cells, miR-200c is encapsulated with cholesterol, an integral part of the biological membranes with very important physical properties of the vehicle. Nano miR-200c showed efficient cellular uptake in KW-634, 821-T4, and 821-LN cells with important changes in gene expression and new isoforms. In KW-634, when treated with encapsulated miR-200c and compare to the non-encapsulated control; miR-29b increased by 5261-fold, and in 821-T4/LN, miR-1247 increased by 150-fold. Conversely, miR-1247 and miR-675 decreased by 348 and 1029.5-fold, respectively. miR-189 decreased by 34-fold in treated 821-T4 cells. A reduction of growth was observed only after 48 h of treatment with Nano miR-200c. Moreover, labeling the vehicle with carboxy-fluorescein showed that the encapsulated particles enter the nucleus and mitochondria. Encapsulated miR-200c by entering the cells, the nucleus and mitochondria, trigger changes in cell cycle phases with 4 up to 12 fold percentage in G2 and S phase respectively compare to miR-200c. Endogenous expression of Nkx2.1, miR-200c, and their targets Myb, Nfib, Six4 and Six1 showed an inverse correlation, as observed in development.

**Conclusions:**

Little is known about miR-200c involvement in regulatory processes. Nano miR-200c affects invasion and migration mechanisms. The expression of encapsulated miR-200c contributes to the inhibition/activation of Kras, EMT, Hippo, regulatory pathways and blockers of metastasis. Delivery of miR-200c increases the expression of miR-29b, an EMY regulator, and miR-1247, an inhibitor of cancer genes, both tumor suppressors involved in lung metastasis. Encapsulated miR-200c act on different proteins that regulates cell cycle pathways. These findings represent a part of a regulatory network providing new insights towards improvement of therapy.

**Electronic supplementary material:**

The online version of this article (10.1186/s12885-019-5337-6) contains supplementary material, which is available to authorized users.

## Background

One of the main mechanisms of gene silencing is controlled by new biomarkers called microRNAs, which are small noncoding RNAs capable of regulating target mRNAs associated with tumors [1, 2]. MicroRNA-mediated gene silencing has emerged as a major mechanism of gene regulation by controlling in a cell and tissue-specific manner, the translation and stability of target mRNAs, thereby controlling the proper balance of proliferation, invasion, migration, differentiation and cell death [3, 4], playing a major role in the inhibition, development and progression of lung cancer. A wide spectrum of disorders, such as pulmonary diseases, diabetes and cardiac disorders, can be attributed to miRs dysfunction [5]. miR-200c-3p is crucial in acute respiratory distress syndrome [6]. microRNA-200c, a known tumor suppressor, is correlated with expression of the transcription factor Nk2 homeobox1 (Nkx2.1) in mouse primary lung cancer cells and early stage mouse lung embryos [7]. Nkx2.1 is also known to suppress lung adenocarcinoma progression [8]. Despite the high prevalence and poor prognosis of lung cancer, little is known about the mechanisms of progression and pathogenesis, or how to interfere with them for novel therapeutic interventions. Current therapeutic options for advanced lung cancer, if available, remain largely ineffective [9, 10], and it is still unclear whether cancer cell invasion or metastasis is the main cause of mortality. In this study, we investigated the targeting ability of miR-200c within our formulation vehicle to demonstrate its regulatory and therapeutic capabilities, as the use of carriers to deliver drugs or genes into cells has been proven to be a successful strategy to increase specificity in targeting cancer cells. We describe a new mechanism of uptake of encapsulated Nkx2.1-dependent miR-200c that can block cells by preventing them from invading surrounding tissues and terminating growth. This formulation has a potential therapeutic role in lung cancer with efficiency for new gene and protein expression, as encapsulation protects miRNAs from nuclease degradation and leads to long-lasting vehicle targets and a systemic presence, which enhances stimulation of cells from the cytoplasm to the nucleus and mitochondria, the center of pro and antiapoptotic pathways.

## Introduction

Lung cancer, a leading deadly disease in men and women with hundreds of new cases per year [11], has developed resistance to generic drugs over time, and microRNAs recognized as key players in the development of this malignancy, could provide potential treatment options. miR-200c functions as a tumor suppressor gene by inhibiting epithelial-to-mesenchymal transition (EMT) during tumor invasion, migration and metastasis. A multiplex network of pathways governs miR-200c and its down regulation is the main mechanism in cancer and many solid tumors [12–16]. Previous studies have identified a regulatory link between miR-200c and Nkx2.1, the developmental and oncogenic transcription factor Six4, a biomarker involved in non-small cell lung tumor initiation and progression [17, 18], Nfib and Myb, with roles in the regulation of gene expression, control of carcinogenesis and cellular senescence [7], as well as important signaling pathways and molecules [19]. These transcription factors [20, 21] and other known miR-200c targets, such as E2F3 and Kras, have been linked to lung epithelial proliferation in development acting as oncogenes in tumorigenesis [22]. Myb has recently been linked to the differentiation of airway epithelial cells and is regulated by miR-200c in glioblastomas [23] and breast cancer cells. Tumor growth can be reduced by varying the expression of Nkx2.1 and the amount of miR-200c in the cell. The delivery of genes into cells, specifically to the nucleus, is problematic due to the specificity of this entity. The effectiveness of a drug delivered in a Nano vehicle compared with the drug itself has been proven to be increased in a xenograft mouse epidermoid carcinoma model [24], as well as for delivery of genes or a combination of genes and drugs together.

Small noncoding RNAs called miRs are “a new” class of tissue-specific genes that function in the development of many organs and a host of human diseases such as cancer through post-transcriptional gene regulation. miRs in animals and plants target mRNAs in a regulatory manner for cleavage or translational repression, making them appropriate for tumor and other disease classifications and providing prognostic and diagnostic targets [3, 25]. In a previous study in which miR-200c was highly expressed while Nkx2.1 was knocked down, the active delivery of miR-200c increased by *16.7*-fold [26], especially when delivered in a Nano vehicle, because miR-200c has been previously shown to enhance radio sensitivity with an antitumor activity drug association in lung cancer [27, 28]. We have recently reported the first evidence that Nkx2.1 regulates a number of miRs, including miR-200c, which mediates gene silencing induced by Nkx2.1 [7]. Here, we determine the efficacy of the treatment of cells with miR-200c compared with miR-200c encapsulated in a Nano vehicle to target the nuclei of the primary mouse lung cancer cells associated with EMT, KW-634, 821-LN and 821-T4. Several microRNAs control tumor cell proliferation, invasion, and survival [29, 30]. The dysregulation of normal patterns of miR-200c expression occurs in multiple types of cancer cells and is linked to tumor progression [22]. Although miRs regulate diverse functional cellular processes, lung transcription factor networks are not yet established, and little is known about their regulation and connection, prompting us to study the dynamic interactions among cancer cells, Nkx2.1, their regulated miRs, the associated tumor microenvironment and the subsequent influences on disease initiation, growth, and spread. These features are critical for determining new therapeutic options for advanced lung cancer associated with the metastatic cascade [31].

Nanotechnology is the science and engineering of vehicles and carriers at the nanoscale, and our preparations are less than 100 nm in size and exhibit great stability up to more than 6 months at 4 °C. In the biological field, a Nano vehicle is a promising way to deliver drugs or genes into cells by increasing their cellular entry and composition by resembling the bilayer phospholipid membrane. By developing a Nano delivery vehicle *also used as empty control* overexpressing miR-200c as a novel strategy to attack lung cancer cells, we further suppressed invasion and migration compared to miR-200c non-encapsulated showing increase levels of miR-29b, a target miR for lung cancer treatment [32, 33], and miR-1247, an inhibitor of key cancer-promoting genes, by encapsulating stable specific amounts with higher cellular uptake. Reduction and alteration of miR-200c are known to trigger cancer progression and genesis by their interactions with various cellular signaling molecules and important pathways. miR-200c is an immunotherapy target against various cancers [34]. In recent years, multiple miR-based drugs have been explored and have entered clinical testing [23, 24]. These studies support our findings that the use of a stable Nano miR-200c increases the efficacy and specificity of delivery in cells and, depending on the expression of Nkx2.1, could be a promising pathway to target cells for cure and potential diagnostic prior to treatment. Interestingly, cancer cells treated with Nano miR-200c express more mitochondrial RNAs (mtRNAs) than non-Nano miR-200c, providing novel insights into the roles of mitochondria with important clues to *understand many* molecular mechanisms, cellular apoptosis and cell death. Our delivery system-based therapeutic miR-200c assumes new strategies to circumvent cancer challenges by efficiently delivering a stable miR-200c that reduces cell growth, invasion and migration, as well as the expression of key oncogenes and protein targets. Using lung cancer cell lines, we demonstrate that Nkx2.1 and its targets, miR-200c, miR-29b, miR-1247, and miR-189, are parts *of a newly discovered* regulatory network that is important for the exploration of new therapeutic approaches in primary and metastatic lung cancer.

## Methods

### Nano formulations, size measurements and zeta (ζ) potential

The vehicle for emulsification was prepared by weighing L-α-phosphatidylcholine, hydrogenated soy (HSPC) (Avanti Polar Lipids, 840,058), 1,1-distearoyl-sn-glycero-3-phosphoethanolamine-N-[methoxy(polyethylene-glycol)-2000] (Avanti Polar Lipids, 880,120) and cholesterol (Sigma) in a sonication vial. PBS (1.5 mL) was added to the dry materials and exposed to ultrasound for 20 s. PBS was further added to fill the vial, and then the vial was exposed to ultrasound for 30 min. The Nano emulsion of the vehicle was prepared from the suspension using a microfluidizer processor model M-110 L (Microfluidics Corporation, Newton, MA). Microfluidizer processors provide high pressure and a resultant high shear rate by accelerating the product through microchannels to a high velocity for size reduction to the nanoscale range. The fluid was split in two and pushed through microchannels with typical dimensions on the order of 75 μm at high velocities (in the range from 50 to 300 m/s). As the fluid exits the microchannels, it forms jets, which collide from opposing microchannels. In the channels, the fluid experiences high shear forces (up to 107 1/s), with orders of magnitude greater than those of conventional technologies. Jet collisions mix at the sub-micrometer level. High shear and impact are responsible for particle size reduction to the Nano range of less than 100 nM with multiphase fluids mixing in the microfluidizer.

The stable, finer Nanoencapsulations of miR-200c were prepared in a sonication vial. PBS (1.5 ml) was added and exposed to ultrasound from a microfluidizer processor model M-110 L powered up to 1241 bar with less than 24 scfm air using a 5.6 kw (7.5 hp) compressor (Microfluidics Corporation, Newton, MA) as previously described [24]. Size measured with the Zetasizer nano series (Malven) by diluting 10 μl of the Nano preparation in 990 μl of PBS. Incorporation of miR-200c (Ambion) in the vehicle is achieve by adding 50 nM of the miR then sonicating the sample in the Fisher Scientific Sonic Dismembrator model 500 for mixture and stability.

Zeta (ζ) Potential: The measurement of ζ potential is based on forming colloidal particles of miRs dispersed in the formulation solutions electrically charged due to their ionic characteristics and dipolar attributes. Each particle dispersed in the solution using the Malvern Zetasizer nano series Zen 3600 (Malvern Instruments Ltd., Enigma Business Park, Grovewood Road, Malvern, Worcestershire WR14 1XZ, U.K.) is surrounded by oppositely charged ions called the fixed layer. Outside the fixed layer, there are varying compositions of ions of opposite polarities, forming a cloudlike area. This area is called the diffuse double layer, and the whole area is electrically neutral. When a voltage is applied to the solution in which the particles are dispersed, particles are attracted to the electrode of the opposite polarity, accompanied by the fixed layer and part of the diffuse double layer, or internal side of the sliding surface. This system uses dispersion technology software (DTS v4.20), which changes to the ζ measuring mode, and the sample for which ζ is measured is contained in disposable capillary cuvettes (DTS1060) equipped with electrodes. Each determination was performed in triplicate.

### Cell lines

Primary non-metastatic and metastatic tumor cells, KW-634, 821-LN and 821-T4, were grown in RPMI + GlutaMAX -1 (1X) (Gibco for Life Technologies REF: 61870–036). The cells were grown in culture medium to reach 40–50% confluency before treatment with 10 μl of 50 nM Nano-miR-200c, miR-200c, empty Nano Control (same composition as the suspension or vehicle with no miRs), antago-miR or control untreated plates before harvesting after 48 h to study the effect of our treatment.

### Characterization and localization of Nano miR-200c by immunofluorescence (IF)

Cells were seeded on square coverslips (Fisher scientific 12–545-80 12 CIR-1) in six-well plates and transfected with 50 nM of each a) naked miR-200c, b) Nano fluorescein miR-200c, c) empty Nano control (same composition as the suspension or vehicle with no miRs), or d) nontreated control. For nuclear staining, molecular probes Prolong® Gold antifade reagent and 1 mg/ml 40,6-diamidino-2-phenylindole (DAPI) were dropped onto the cells for 10 min, after which the cover glass was flipped upside down, with cells facing the slide, and sealed with nail polish after the Prolong® had dried. The cells were washed three times with PBS following each step of the staining procedure with images obtained using a fluorescence microscope Leitz Aristoplan (WILD LEITZ GMBH) at high magnification of 60X under oil.

### Cell cycle analysis for viability and cytotoxicity

Cell cycle was analyzed by flow cytometry after the addition of propidium iodide. Cells *non treated control*, treated with Nano miRs (200c, 221, 222, 346, and 1195) and empty Nano negative control (same composition as the suspension or vehicle with no miRs)were washed with cold PBS, resuspended in PBS, fixed by addition of equal volume of cold absolute ethanol and incubated for two or more hours at 4 °C. Cells were then washed with cold PBS, stained with propidium iodide (0.1% Triton X-100 and 0.2 mg/mL DNAse-free RNAse A in cold PBS) and stored at − 80 °C before FACS for data acquisition by flow cytometry.

### Real-time qRT-PCR

miR expression was analyzed by RT-qPCR in total RNA samples using methods described previously [7]. Per sample, 10 ng to 1 μg was subjected to reverse transcription using TaqMan RNA and MicroRNA Reverse Transcription Kits (Applied Biosystems, Grand Island, NY), respectively. qRT-PCR analysis of RNA and miRNAs was performed in triplicate with TaqMan RNA and MicroRNA Assays (Applied Biosystems) according to the manufacturer’s instructions. GAPDH and RNU6B were selected for normalization. Each experiment was performed in triplicate, and quantitative analysis of changes in expression levels were calculated by StepOne Real-Time PCR System (Applied Biosystems). miR expression was analyzed by qRT-PCR using total RNA. Quantitative analysis was performed by the 2^-ΔΔCt^ methods.

### Mitochondrial membrane potential assays

We used a fast, less toxic and convenient CellLight® Mitochondria-RFP, BacMam 2.0, which provides an easy method for labeling mitochondria with Red Fluorescent Protein (RFP) in live and fixed cells. The reagent was added to the cells, incubated overnight, and the cells imaged. This ready-to-use construct was transduced and transfected into cells using BacMam 2.0 technology, where it expressed RFP fused to the leader sequence of E1 alpha pyruvate dehydrogenase. Cells were treated with 10 μl of miR-200c or Nano miR-200c for 24 to 48 h, followed by 12 μl of JC-9 dye mitochondrial probes RFP, Bachman 2.0 (applied BioSystems) per the manufacturer’s instructions for another 24 h. They were then fixed with formaldehyde before visualization under a fluorescence microscope.

### Small RNA and RNA sequence preprocessing and filtering data

Each sequencing run used a 150 or 300 cycle NextSeq 500 v1 or v2 High Output Sequencing Kit, and corresponding High Output Flow Cell. Illumina libraries were quantified using qRT-PCR. Loading concentrations varied between 0.8 and 1.7 pM. The paired-end library was enriched before being sequenced on the Illumina HiSeq at the WIBR and MIT BioMicro Center. The location of each barcode was recorded, and this allowed for the post-sequencing identification of each population. Accession number GSE125000.

### In situ hybridization

We performed in situ hybridization on both frozen sections according to Obernosterer’s protocol (Alenius, et al., 2007). Briefly, E11.5 whole embryos and E19.5 dissected lungs were fixed in freshly prepared 4% paraformaldehyde in 1X PBS, pH 7.4, at 4 °C for 16 h, while left lungs were fixed in 4% PFM at 4 °C overnight followed by sequential washing with DEPC-treated PBS (30 min) and 7.5% sucrose (30 min). Tissues were then transferred to new 50 ml conical tubes containing 50 ml of 30% sucrose dissolved in DEPC-treated PBS and rocked gently in the cold room for 2 days. Tissues were then embedded in OCT using liquid nitrogen, sectioned and mounted on glass slides before being stored at − 20 °C. The same thickness slides were used for ISH. After warming up at RT for 30, the tissue slides were first washed with DEPC-treated PBS (3X 3 min), followed by acetylating in triethanolamine buffer plus acetic anhydride for 10 min, permeabilizing in PBST (PBS plus 0.1% Triton X-100 in DEPC-treated water) for 30 min, and washing 3X 5 min at RT. After prehybridization at RT for 2 h, hybridization was carried out at 50 °C overnight in the same prehybridization buffer (50% formamide, 5x SSC, 1x Denhardt’s solution, 500 μg/mL salmon sperm DNA, and 5% dextran sulfate) containing 25 nM of miR29b locked nucleotide acid, Exiqon (LNA) probe (Alenius, et al., 2007), followed by sequential washing with 0.2x SSC at 50 °C for 1.5 h, cooling down to RT, and washing again with 0.2X SSC for 5 min and PBS for another 5 min at RT. The slides were then incubated in blocking solution (TTBS, 0.05 M Tris, pH 7.5, 0.15 M NaCl, 0.1% Tween-20, and 5% sheep serum) at RT for 1 h before incubation with anti-digoxigenin-AP antibody (1:2500, Roche) overnight at 4 °C. After washing in TTBS for 3X 10 min, the slides were incubated at RT for 10 min in 50 ml color reaction buffer (0.1 M Tris, pH 9.5, 0.1% Tween-20, 0.1 M NaCl, and 0.05 M MgCl2) before color development with BM purple (Roche) plus 0.1% Tween-20. The sections were air-dried and cover slipped with Prolong Gold (P36935, Invitrogen), and images were obtained using a LSM 510 Axiovert 200 M.

### Migration and invasion assays

Cell migration and invasion were performed using the Cytoselect 24-well cell migration assay (8 μm, Fluorometric Format) (Cat. No. CBA-101-C) from Cell Biolabs. Approximately 24 to 48 h after transfection and exposure to Nano miR-200c, cells in serum-free medium were seeded into the upper chambers of the transwells, which were lined with Matrigel (BD Biosciences), for the migration (8 mm pore size, Costar) and invasion assays. The lower chambers were filled with medium containing 10% FBS. After several hours of incubation at 37 °C with 5% CO2, the cells that had migrated or invaded through the membrane were fixed using formaldehyde and stained with 0.1% crystal violet. The cells on the lower surface were photographed, and five random fields of cells were counted. Three independent experiments were performed.

### Protein insights into signaling pathway activation

The ActivSignal IPAD Platform was used to help understand the pathway activation/inhibition profile of cells by assessing the signaling pathways in each sample. The IPAD assay allows the direct measurement of the expression and modification of signaling pathway components. Here, we focused on multiple targets reflecting the activities of major signaling pathways in our sample lysates (treated vs. untreated or treated empty Nano control). The samples were processed, and target proteins were detected with content analysis.

### Statistical analysis

For all experiments, statistical analysis was first performed using one-way analysis of variance (ANOVA) to determine statistical significance between groups for each endpoint assessed. To assess the in vitro oncolytic effects, statistical significance between groups was calculated with multiple comparisons between treatment groups and controls evaluated using Student’s t-test. All *p*-values < 0.05 were considered statistically significant. For mRNA NGS data analysis, principal component analysis (PCA) was used in the unsupervised analysis to reduce the dimensions of large data sets and to explore sample classes arising naturally based on the expression profile. Our NGS data (Accession number GSE125000) analysis pipeline is based on the Tuxedo software package, which is a combination of open-source software and implements peer-reviewed statistical methods. In addition, we employed specialized software developed internally at Exiqon to interpret and improve the readability of the final results. The components of our NGS data analysis pipeline for RNA-Seq include Bowtie2 (v. 2.2.2) [35], TopHat (v2.0.11) [36] and Cufflinks (v2.2.1) [37, 38].

## Results

### Formulation, ζ potential, and particle size of empty Nano and Nano miR-200c

**Fig. 1:**
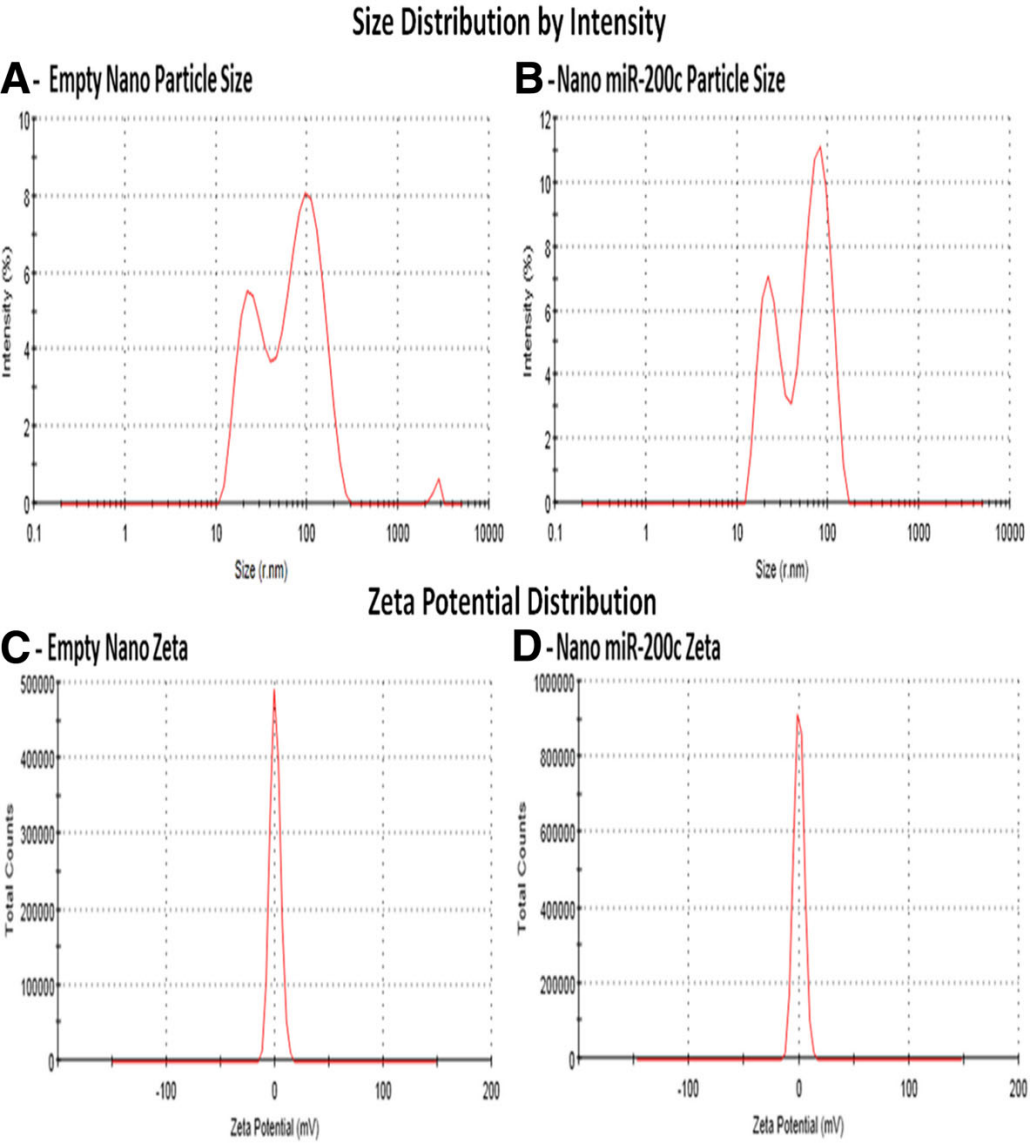
Dynamic laser light scattering particle size analysis of our empty Nano *control without any miR* and Nano miR-200c showing the Z-average size distribution of the particle and micro-fluidization results, with a dramatic decrease in particle size, and demonstrating the heterogeneity of particle size, even within what appears to be a homogeneous distribution. **a**- The size of our empty Nano vehicle; **b**- The size of the vehicle plus miR-200c; **c**- The Zeta potential of empty Nano; and **d**- The Zeta potential of the Nano miR-200c encapsulated gene

The particle size Z-average of the empty Nano *vehicle used as a control for paralleled treated cells* and Nano miR-200c suspension was detected by the Malvern particle size analyzer and subsequently reduced to 48.58 and 55.50 nm respectively after micro-fluidization. The size of the Nano miR-200c particle with incorporation of carboxy-fluorescein and miR-200c was 48.76 nm (Fig. 1a). The interaction of miR-200c with our formulation process decreased the ζ potential from 0.212 to − 0.075 for 100% of the area (Fig. 1b). Our delivery system-based therapeutic miR-200c allows new strategies to circumvent cancer challenges by efficiently delivering a very stable miR-200c that can persist for more than 6 months if not than a year of the active pharmaceutical ingredient (Additional file 1: Figure S1) *with the same pharmacokinetic properties*.

### Efficient miR-200c localization, cellular uptake in cells and the cell cycle

**Fig. 2:**
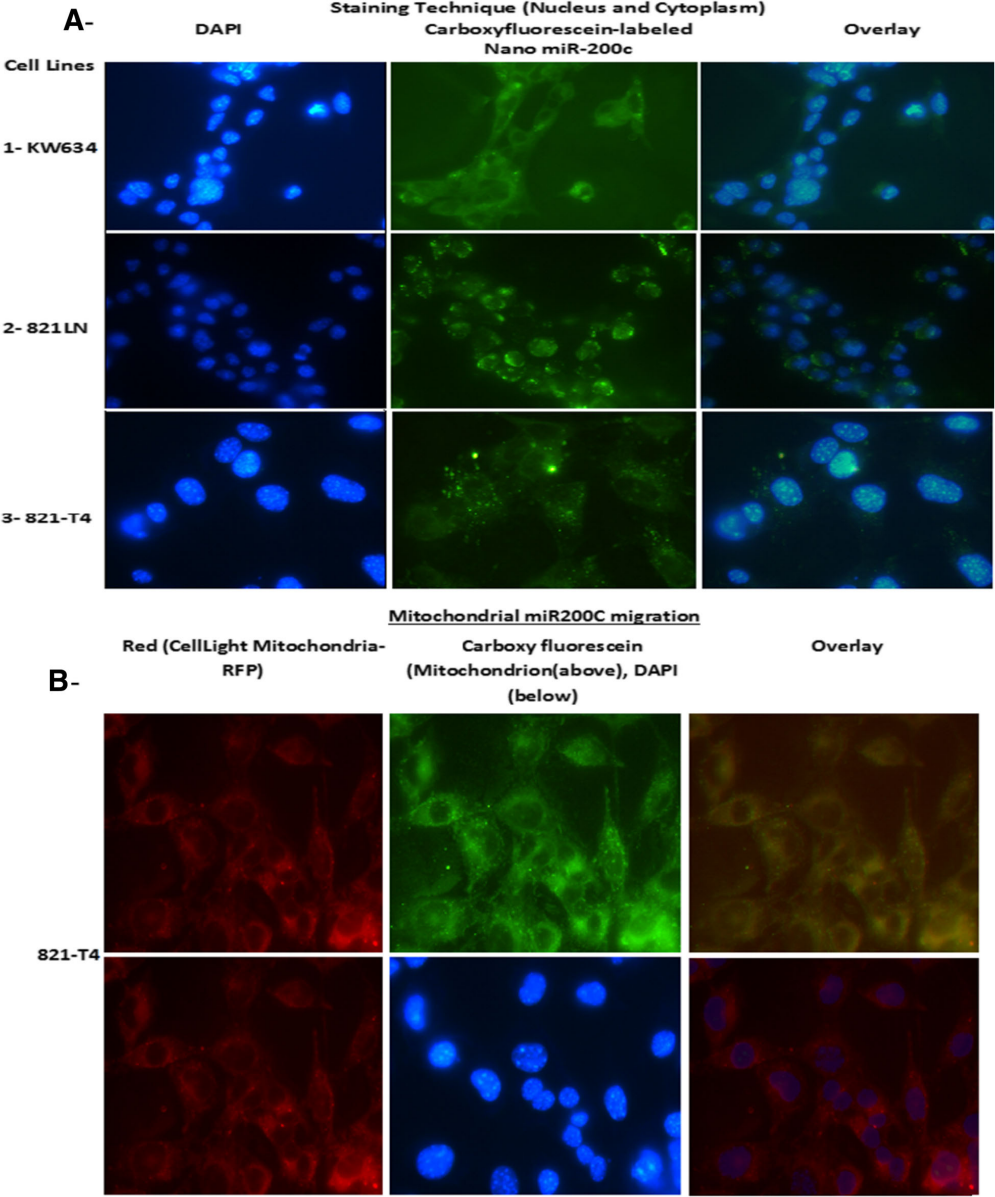
**a**- Efficient cellular uptake of miR-200c carboxy-fluorescein-labeled nanoparticles. Confocal microscopy analysis of the cellular uptake of liposomes tagged with fluorescein 24 h after transfection with 100 nmol/l (A1) 95% of miR-200c enter KW-634 non-metastatic lung tumor cell line, (A2) miR-200c in the 821-LN metastatic tumor cell line, and (A3) miR-200c in the 821-T4 metastatic tumor cell line. **b**- Staining techniques showing cytoplasmic and nuclear carboxy-fluorescein-labeled incorporation of our Nano miR-200c and Red CellLight Mitochondria-RFP miR-200c migration in the 821-T4 metastatic tumor cell line at about 87.5%, allowing the observation of the mitochondrial response to our gene transfer

**Fig. 3:**
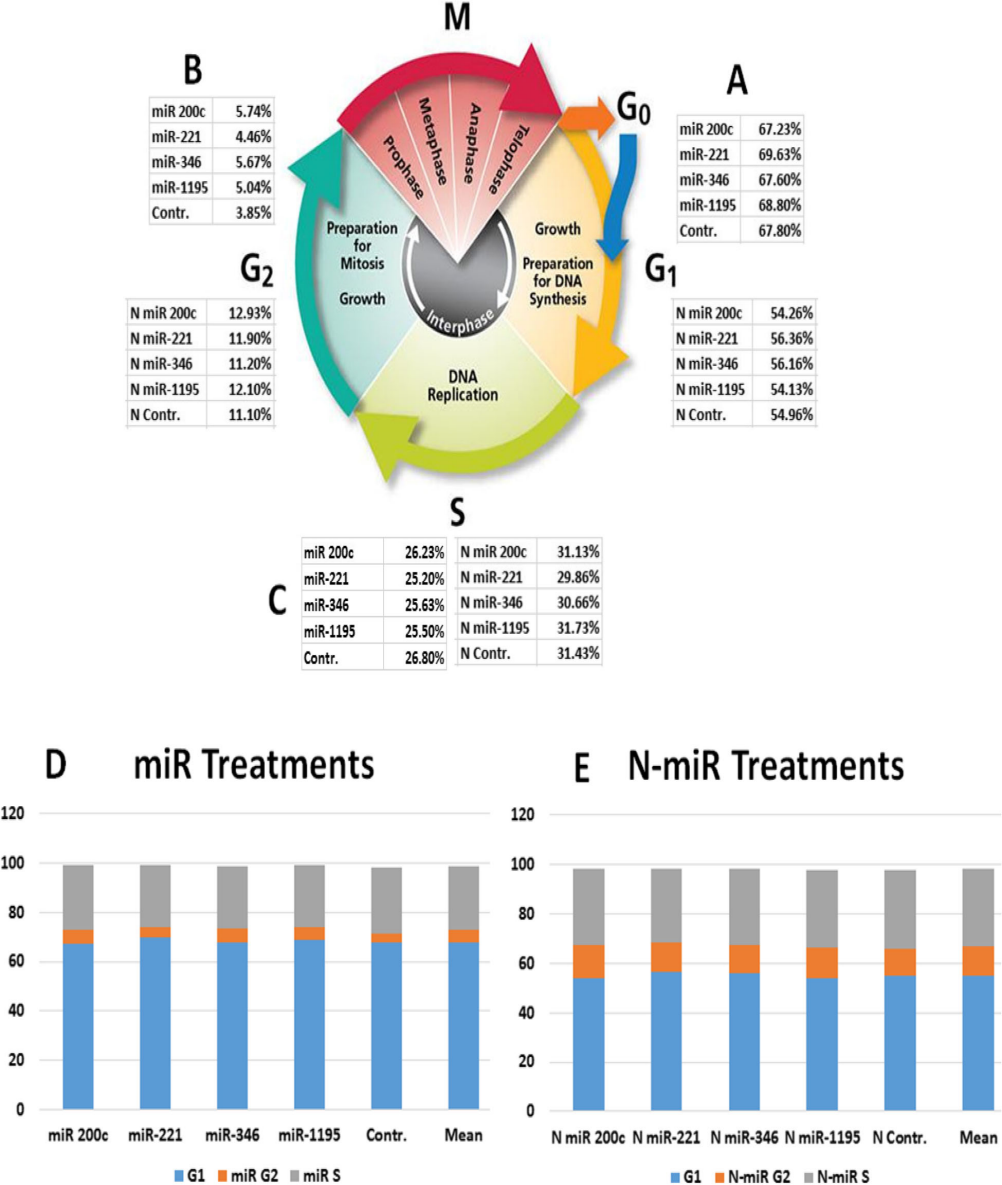
Cell cycle phases (**a** at G1, **b** at G2 & **c** the S phase) and analysis by flow cytometry with location of miRs (200c, 221,346 and 1195) after treatments (**d**) with miRs and (**e**) with N-miRs *in addition to the control without any miR and non-treated control cells*

Confocal microscopy was performed to determine the localization of our Nano formulation with 87.5 to 95% of our particles entering the cells after they were incubated with carboxy-fluorescein-labeled cholesterol and 1,2-disteroyl-sn-glycero-3-phospho-ethanolamine encapsulated with miR-200c for 24 h after transfection with 100 nmol/l miR-200c in the KW-634 non-metastatic lung tumor cell line (Fig. 2 A_1_), miR-200c in the 821-LN metastatic tumor cell line (Fig. 2 A_2_), and miR-200c in the 821-T4 metastatic tumor cell line (Fig. 2 A_3_). Staining techniques revealed cytoplasmic and nuclear carboxy-fluorescein-labeled incorporation of our Nano miR-200c and Red CellLight Mitochondria-RFP miR-200c migration in the 821-T4 metastatic tumors cell line, allowing us to observe the mitochondrial response to gene transfer (Fig. 2b). Interestingly, the nanoparticles are localized in the cytoplasm of the cells, in the nucleus or close to its membrane and in the mitochondria (Fig. 2a and b). Cell cycle FACS analysis of various encapsulated miRs (200c, 221, 222, 346, and 1195) with negative empty vector control (same composition as the suspension or vehicle without miRs) was performed to examine the viability and cytotoxicity of the Nano preparation by flow cytometry after addition of propidium iodide. There is a significant change in cell cycle phases after the treatments by encapsulated miR-200c compare to miR-200c (Fig. 3 and Additional file 1: Figure S2). Encapsulated cells entering the preparation for DNA synthesis G1 phase of this naturally occurring cell proliferation process are 2 to 4 fold changed when preparing for Mitosis in G2 and 6 plus fold higher when replicating in S phase pointing to very interesting regulatory mechanisms as our formulation presents an hydrodynamic size and indication that their robust limited size affect the downstream process.

### Effects of encapsulated miR-200c on the growth, morphology and expression of its endogenous targets

**Fig. 4:**
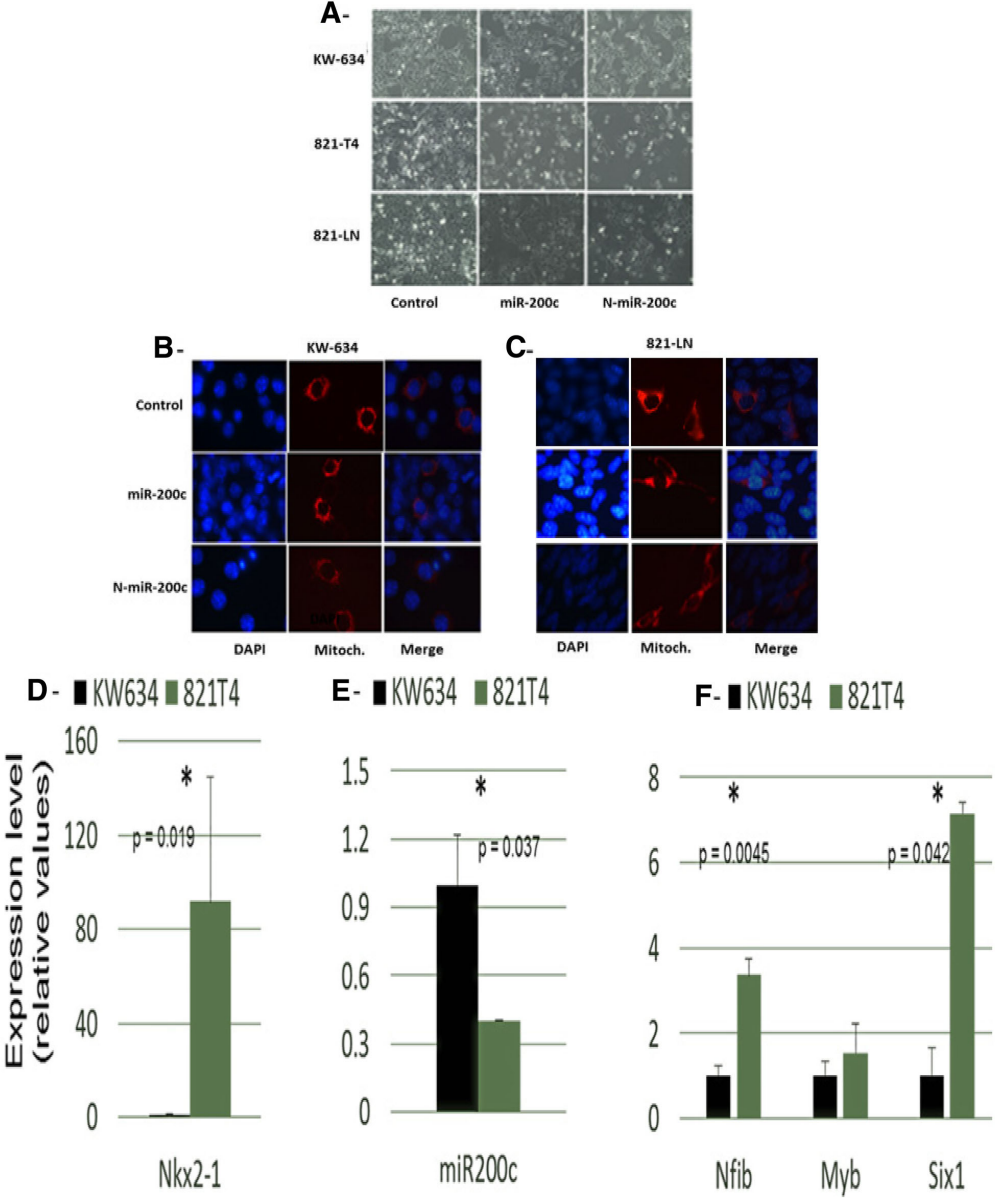
**a**- Effect of the treatment on the cancer cell lines. From left to right: untreated cells, cells treated with miR-200c, and cells treated with Nano miR-200c in mouse lung non-metastatic (KW-634), metastatic 821-LN and metastatic 821-T4 cell lines, showing a reduction of cell growth particularly following treatment with Nano miR-200c. **b**- and **c**- Cancer cell features after transduction with cell light Bacman reagent. From left to right: DAPI, mitochondrial membrane potential staining, and correlation showing the efficiency of cellular uptake of Nano miR-200c carboxy-fluorescein-labeled nanoparticles in mouse lung non-metastatic (KW-634) and metastatic (821-LN) cell lines, demonstrating a significant growth reduction with no change or damage in the nucleus or mitochondria. **d**- Endogenous expression of Nkx2–1, **e**- miR-200c and **f**- its targets Myb, Nfib and Six1 in mouse lung non-metastatic (KW-634, black bars) and metastatic (821-T4, green bars) cell lines, showing the same inverse correlation as described in MLE15 cell line. The asterisk indicates statistical significance

To determine whether Nano miR-200c could directly affect the growth of tumor cells more than miR-200c, the effect of Nano miR-200c treatment on the growth of tumor-derived cell lines from tumors isolated from Kras/Lkb1 was examined. A greater reduction in growth could be observed in cells treated with the Nano miR-200c compared with untreated cells after a prolong time. The non-metastatic KW-634 and metastatic 821-T4 and 821-LN cells (Fig. 4a) treated with miR-200c particularly encapsulated miR-200c are narrower than normal or nontreated cells. CellLight® Mitochondria-RFP, BacMam 2.0 provided a way to label mitochondria with red fluorescent protein (RFP) in live cells. This reagent is transfected into cells using BacMam 2.0 technology, where it expresses RFP fused to the leader sequence of E1 alpha pyruvate dehydrogenase. We observed mitochondria-RFP behavior in live cells independently of the mitochondrial membrane potential. This process allowed us to observe some mitochondrial disorders in the cytoplasm of some cells. Those disorders were present in untreated cells as well as those treated with miR-200c and Nano miR-200c, thus confirming that it was not related to our treatment (Fig. 4b and c). Endogenous expression of Nkx2.1 (Fig. 4d), miR-200c (Fig. 4e), and its direct targets Myb, Nfib and Six1 (Fig. 4f) in mouse lung non-metastatic (KW-634, black bars) and metastatic (821-T4, green bars) cells showed the same inverse correlation as observed in MLE15 cells (*) *P* < 0.05. To characterize these cells, we used qRT-PCR to evaluate endogenous expression levels of Nkx2.1, miR-200c, and the downstream targets Myb, Nfib, Six4, and Six1 that we had previously shown to be part of a regulatory network in MLE15 cells and in Nkx2.1 mutated lungs. Higher levels of Nkx2.1 were detected in 821-T4 metastatic compared with non-metastatic KW-634 cells. In these cells, we found an inverse pattern of expression between Nkx2.1 and miR-200c and an inverse pattern between miR-200c and downstream targets Myb, Nfib, Six4, and Six1 (Fig. 4) as they are all direct targets of Nkx2.1.

**Fig. 5:**
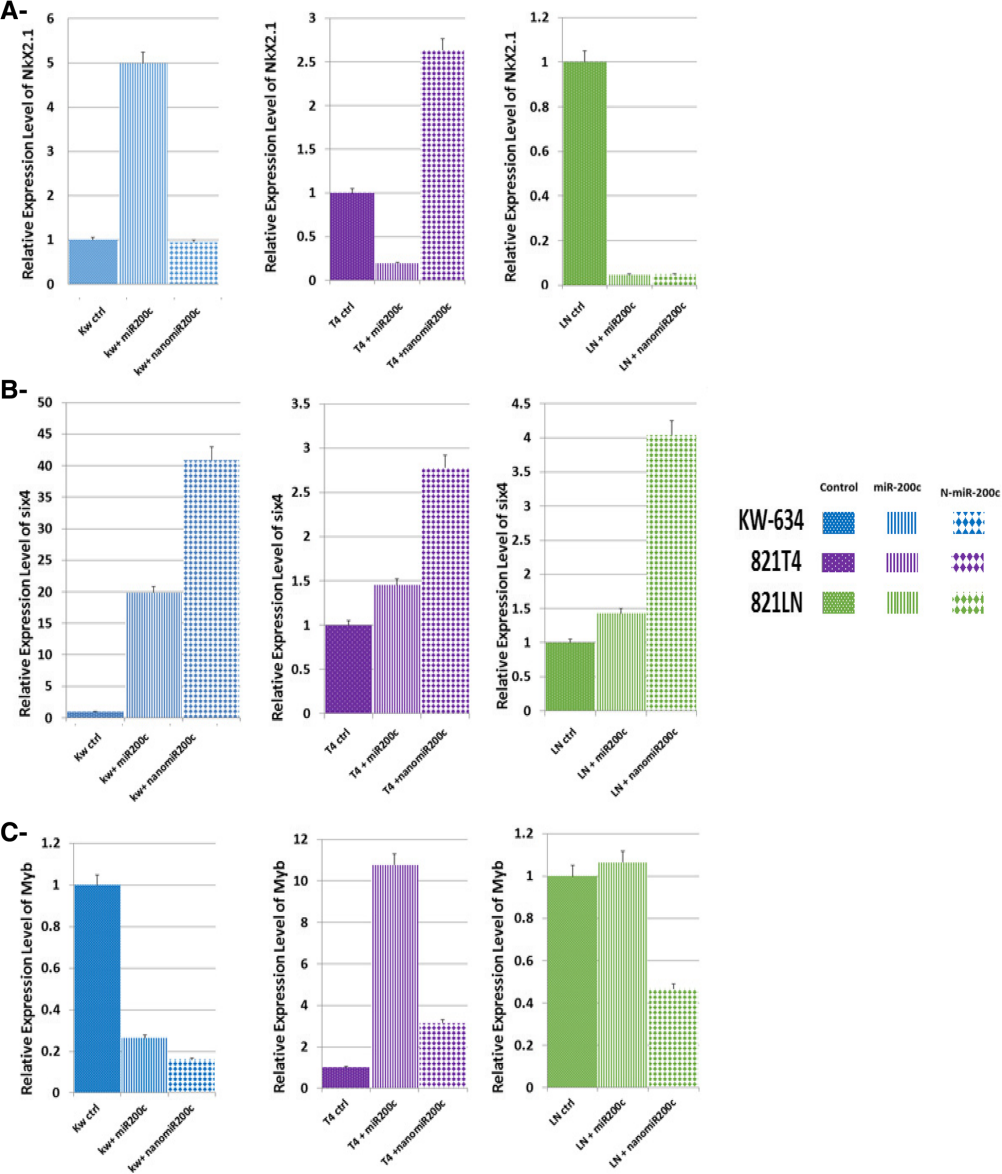
Endogenous expression of direct targets of miR-200c in mouse lung non-metastatic (KW-634, blue bars) and metastatic (821-T4, violet bars; 821-LN, green bars) cell lines, showing the various roles and effectiveness of our Nano encapsulation of miR-200c after each treatment with 50 nM miR-200c and nano-miR-200c. **a**- Nkx2.1 expression levels were maintained following treatment with Nano miR-200c in non-metastatic KW-634 and were elevated in metastatic 821-T4, while they were almost undetectable in metastatic 821-LN. **b**- Six4 showed significant expression in the cell lines treated with miR-200c and Nano miR-200c, and **c**- Myb expression was considerably affected by Nano miR-200c treatment in all lines

To clarify the significance of miR-200c in the metastatic and non-metastatic cell lines, we used qRT-PCR to investigate the effects of the expression of miR-200c and Nano miR-200c on some of its known direct targets (Fig. 5). The level of Nkx2.1 in KW-634 cells treated with Nano miR-200c was closely the same as in untreated controls, while miR-200c-treated cells expressed 5-fold higher levels than the controls. Within 821-T4 metastatic cells, Nano miR-200c-treated cells had a 2.5-fold higher expression of Nkx2.1 expression compared with the controls, while miR-200c-treated cells expressed 2-fold lower levels than Nano miR-200c. In 821-LN metastatic cells, treatment with miR-200c and Nano miR-200c had the highest downregulatory effect on the expression of Nkx2.1 compared to controls (Fig. 5a). In non-metastatic KW-634, metastatic 821-T4, and metastatic 821-LN cells, treatment with miR-200c and Nano miR-200c upregulated Six4 expression by 20 to 40, 1.4 to 2.75 and 1.4 to 4.4-fold, respectively, compared to the control (Fig. 5b). Myb expression is downregulated by 3.6 and 4.3-fold when non-metastatic KW-634 cells were treated with miR-200c and Nano miR-200c, respectively, compared to controls. In 821-T4 metastatic cells treated with miR-200c and Nano miR-200c, expression of Myb was 11 and 3-fold higher, respectively, compared with the controls. Myb expression in 821-LN metastatic cells was not significantly altered between controls and miR-200c treated cells but decreased by 50% following treatment with Nano miR-200c (Fig. 5c). miR-200c when encapsulated are being potential very important therapeutic targets as they can inhibit or activate signaling pathways as modulating cellular events as what we have seen in the cell cycle previously described.

### NGS of mRNAs and miRs after treatment with miR-200c and encapsulated miR-200c

**Fig. 6:**
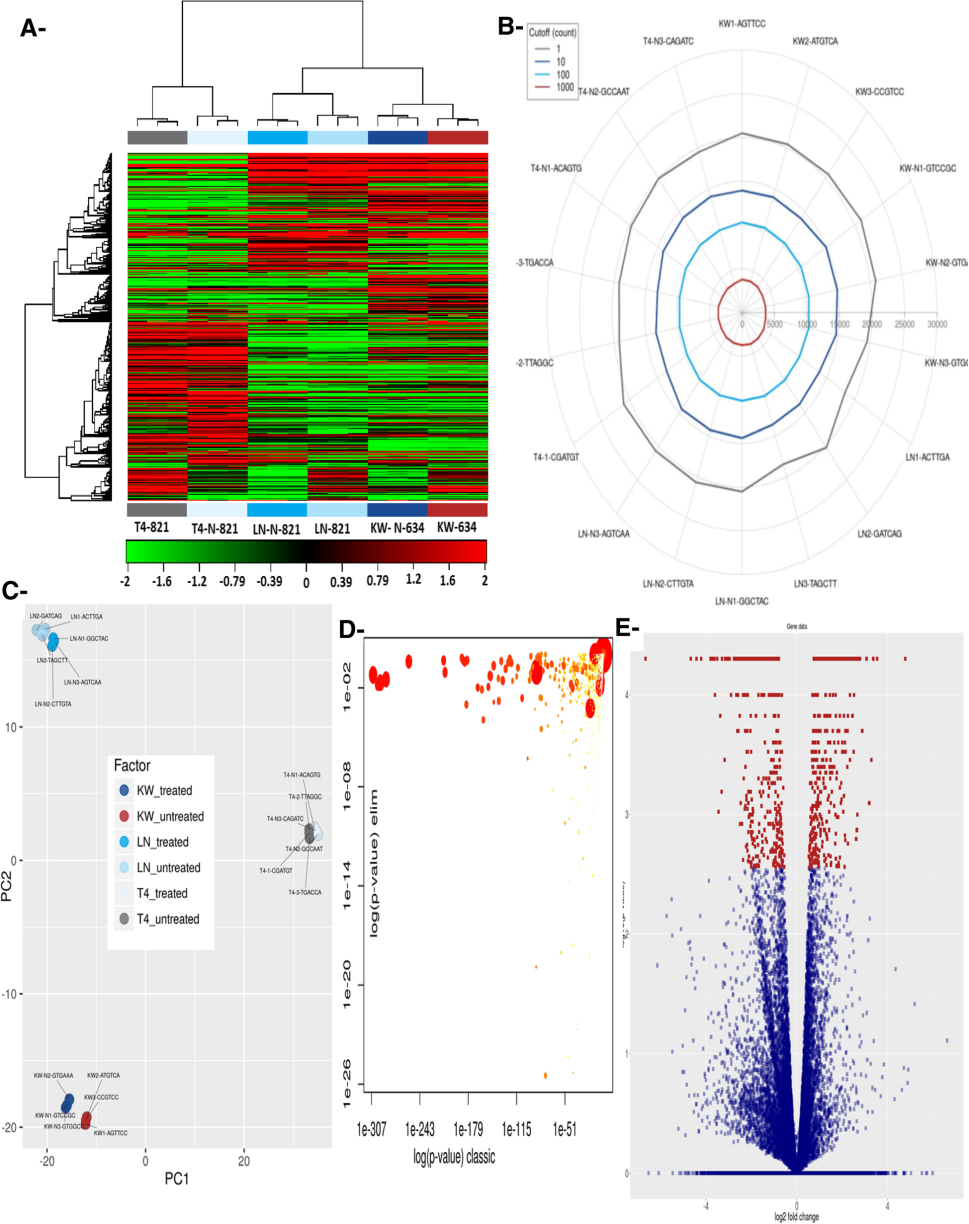
**a**- Heat map and unsupervised hierarchical clustering by sample and genes were performed for the listed samples using the 500 genes with the largest coefficient of variation based on FPKM counts. Data were based on samples from the treated and untreated groups. The results were based on the normalized FPKM (abundance) for each gene for each sample and a **b**- radar plot showing the number of genes identified for each sample at different fragment count cut-off values. See the color scale at the top of figure for the specification of fragment count cut-off values. **c**- Principal component analysis (PCA) plot for treated and untreated cells. PCA was performed on all samples that passed QC using the 500 genes with the largest coefficient of variation based on FPKM counts. Each circle represents a sample. Treated KW cells were separated better than 821-T4, which was not well separated, and 821-LN, which was moderately separated. **d**- Scatter plot showing significantly enriched GO terms associated with genes that were differentially expressed between treated and untreated cells. The plot shows a comparison of results obtained with the two statistical tests used. Values along diagonal line were consistent between both methods. Values on the bottom left of the plot correspond to the terms with the most reliable estimates using both methods. The size of a dot is proportional to the number of genes mapping to that GO term, and the coloring represents the number of significantly differentially expressed transcripts corresponding to that term, with dark red representing more terms and yellow fewer terms. **e**- Volcano plot showing the relationship between the *p*-values and the log2-fold change in normalized expression (FPKM) between treated and untreated cells; Data are based on the normalized FPKM (abundance) for each gene per sample

NGS approaches were used for genes identification regulated (up or down regulation) by changes in the expression of miR-200c and comparing expression of these genes following treatment with encapsulated miR-200c and in controls (Fig. 6, Additional file 1: Figures S3 and S4).The two-way hierarchical clustering of the genes and samples with 500 genes heat maps with the largest coefficient of variation were based on FPKM counts. Each row set for a gene, and column set for one sample. Red as the expression levels above the mean and green those below the mean (Fig. 6a) are the relative expression level of a single transcript across samples. We identified genes for each sample based on the count cut-off values of different fragment showing the 20 most differentially important and interesting expressed genes (Table 1).

**Table 1:** The 20 most significantly differentially expressed genes

Gene ID	Gene	Locus	Treated FPKM	Untreated FPKM	Log^2^ Fold Change	q_value
XLOC_001070	Mir29b2, Mir29c, mmu-mir-29b-2	1:194938820–195,037,908	426.25	4.19	−6.67	0.00215219
XLOC_034022	H19, Mir675	7:142575528–142,578,143	20.65	568.45	4.78	0.00215219
XLOC_002346	Gp49a, Lilrb4	10:51480631–51,496,613	24.53	0.97	−4.66	0.00215219
XLOC_024975	–	4:150736119–150,739,106	1.64	0.08	−4.42	0.00215219
XLOC_026432	Car6	4:150187014–150,201,332	3.34	0.18	−4.21	0.00215219
XLOC_036797	Angptl6	9:20868641–20,879,727	28.72	2.07	−3.79	0.00215219
XLOC_013580	AC115005.1, Gm7541	16:8622332–8,624,782	7.85	0.6	−3.7	0.00215219
XLOC_003379	–	10:79038548–79,046,643	3.34	0.26	−3.67	0.00215219
XLOC_002626	–	10:82116731–82,122,868	1.48	0.12	−3.62	0.00215219
XLOC_003263	Col13a1	10:61838235–61,979,108	0.78	0.06	−3.61	0.00382174
XLOC_037321	AC163666.1, SNORD50	9:88595218–88,599,516	1.18	13.74	3,54	0.00215219
XLOC_028771	Fam71f2	6:29275646–29,290,927	28.77	2.54	−3.5	0.00215219
XLOC_008396	–	13:14677687–14,682,438	1.26	0.11	−3.5	0.00215219
XLOC_008544	Dcdc2a	13:25056003–25,210,706	1.56	0.14	−3.44	0.023298
XLOC_012908	Krt6a	15:101689931–101,694,307	3.0	31.08	3.37	0.00215219
XLOC_012225	Soat2	15:102150517–102,163,474	10.76	1.04	−3.37	0.00539903
XLOC_019276	Gm14047	2:129364569–129,376,154	6.63	0.67	−3.31	0.0173413
XLOC_003382	–	10:79048734–79,059,175	1.67	0.17	−3.3	0.00215219
XLOC_002473	Chchd10	10:75935559–75,940,672	62.53	6.36	−3.3	0.00215219
XLOC_036264	2810417H13Rik	9:65828929–65,908,794	4.88	47.75	3.29	0.0105894

Treated and untreated columns show group average FPKM values. FPKM is a unit of measurement of gene expression (fragments per kilobase of transcript per million mapped reads). Transcripts with the highest fold change between groups are shown at the top of the table. The fold change is the log2-fold change of FPKM between the treated and untreated groups. q-values shown are *p*-values that have been adjusted using the Benjamini-Hochberg false discovery rate (FDR) approach to correct for multiple testing. As a general guide, fold changes with q-values below 0.05 may be considered significant

On the outer rim of the plot, the sample names can be found. 1, 10, 100 or 1000 counts estimation of genes identified is illustrated as colored rings. Sample resulting in a significantly lower number of identified genes at each fragment count cut-off was a deviation from the rest. Concentric rings of different colors (Fig. 6b) are the result of genes identified at each fragment count cut-off samples with similar number. Our data revealed that Nano miR-200c increased the expression of miR-29b by 101.7-fold compared with miR-200c treatment (Table 1). In KW-634 cells, miR-29b increased by 5261-fold (Additional file 2: Table S1), and in 821-T4/LN cells, miR-1247 increased by 150-fold. Conversely, miR-1247 and miR-675 decreased by 348- and 1029.5-fold, respectively (Additional file 2: Tables S2 and S3). miR-189 decreased by 34-fold in 821-T4 cells treated with encapsulated miR-200c (Additional file 2: Table S3). NGS comparison of untreated A-KW-634 vs. 821-LN, B-821-LN vs. 821-T4, C-KW-634 vs. 821-T4, treated D-KW-634 vs. 821-LN, and E-821-LN vs. 821-T4 showed completely opposite higher gene expression values compared with the untreated metastatic cells, F-KW/T4 (Additional file 1: Figure S4).

Unsupervised analysis for the reduction of our large data sets was done using Principal component analysis (PCA) according to the expression profiles giving us 500 genes with the largest coefficient on variation plotted along the X-axis and the second largest on the Y-axis according to biological pronounced differences between samples included based on the FPKM abundance estimations included in the analysis for sample clustering. Separation of samples in different regions of a PCA plot are based on their biology. Cells were the main variable factors in addition to the treatments. Clustering was performed according to KW-634 non-metastatic cells, metastatic 821-T4 and 821-LN cells, and their respective treatments (Fig. 6c).

We used Gene Ontology **(**GO) enrichment analysis to investigate whether specific GO terms were more likely to be associated with the differentially expressed transcripts and by comparing two different statistical tests: Standard Fisher’s test to investigate the enrichment of terms between two groups, and the ‘Elim’ method applied to achieve a more conservative approach incorporating the topology of the GO network to compensate for local dependencies between GO that can mask significant GO terms thus highlighting relevant GO terms. Figure 6d is the result for the biological process GO terms of differentially expressed transcripts identified between the two groups. The Volcano plot visualize genes display statistically significant large-magnitude changes with towards the top of the plot (high statistical significance) and at the extreme left or right (strongly down and up-regulated, respectively) by plotting -log10 (*p*-value) on the y-axis and the expression fold change between the two experimental groups on the x-axis with two regions of interest. In red are genes with a q-value < 0.05 with thousands passing the filtering as indicated in Fig. 6e.

The table shows the results for the GO (biological process) terms associated with significant differentially expressed transcripts identified between the two groups. Complete GO enrichment analysis showed all comparisons such as Cellular component (CC) and Molecular function (MF) analysis; the 20 significantly differentially expressed GO (biological process) terms associated with transcripts between the treated and untreated groups were mostly related to Regulation of Transcription and Cell Division as indicated in Table 2.

**Table 2:** Top 20 significant GO (biological process) terms associated with transcripts that were differentially expressed between the treated and untreated groups

GO_ID	Term	Annotated	Significant	Expected	*p*-value
GO:0006355	Regulation of transcription, DNA-template	2829	129	110	3.3e-26
GO:0051301	Cell division	510	96	19.83	5.0e-26
GO:0006281	DNA repair	358	47	13.92	4.6e-25
GO:0007067	Mitotic nuclear division	309	74	13.92	2.1e-23
GO:0006397	mRNA processing	317	23	12.33	4.2e-23
GO:0007049	Cell cycle	1129	167	43.9	1.2e-19
GO:0008380	RNA splicing	254	17	9.88	5.5e-19
GO:0000122	Negative regulation of transcription from RNA polymerase II promoter	539	41	20.96	6.6e-19
GO:0015031	Protein transport	1256	63	48.84	9.5e-19
GO:0006886	Intracellular protein transport	651	38	25.31	2.6e-18
GO:0006364	rRNA processing	111	3	4.32	4.2e-15
GO:0016567	Protein ubiquitination	484	28	18.82	1.7e-14
GO:0045944	Positive regulation of transcription from RNA polymerase II promoter	716	45	27.84	4.5e-14
GO:0006260	DNA replication	232	51	9.02	1.1e-13
GO:0045893	Positive regulation of transcription, DNA template	1074	62	41.76	4.7e-13
GO:0045892	Negative regulation of transcription, DNA template	860	58	33.44	1.8e-11
GO:0001701	In utero embryonic development	371	29	14.43	2.9e-11
GO:0006915	Apoptotic process	1336	91	51.95	6.5e-11
GO:0051726	Regulation of cell cycle	560	82	21.77	1.2e-10
GO:0008285	Negative regulation of cell proliferation	449	30	17.46	2.4e-10

This table aims to highlight the most relevant GO terms associated with the differentially expressed transcripts in the comparison to ascertain whether certain biological functions were enriched among the transcripts compared with the reference background. This analysis is a type of gene enrichment test and does not ensure that the transcripts that belong to a significant GO term are up or downregulated. It does ensure, however, that a group of differentially expressed genes with similar functionality are significantly over-represented. The expected values represent an estimate of the number of transcripts associated with the given significant GO term at random among all the annotated differentially expressed genes. The significant (observed) values represent the number of differentially expressed transcripts associated with that particular GO term in the sample (real) dataset. Annotations represent the total number of genes associated with that GO term in the sample dataset, which means that the reference background could potentially have a larger number of annotations. The q value represents the test statistics for the given GO term whether or not it is significantly enriched

Different isoforms were detected. Novel transcripts that could be a new isoform of a known gene or a transcript without any known features were characterized as transcripts containing features that are not present in the reference annotation (Table 3).

**Table 3:** Isoforms: Table of the 20 most significantly differentially expressed isoforms (known and novel)

Isoform ID	Gene	Locus	Treated FPKM	Untreated FPKM	Log^2^ Fold Change	q_value
XLOC_012369	Ywhaz	15:36767848–36,794,547	1.7	80.48	5.57	0.00942353
XLOC_024975	–	4:150736119–150,739,106	1.64	0.08	−4.42	0.00942353
XLOC_003379	–	10:79038548–79,046,643	3.34	0.26	−3.67	0.00942353
XLOC_002626	–	10:82116731–82,122,868	1.48	0.12	−3.62	0.00942353
XLOC_008396	–	13:14677687–14,682,438	1.26	0.11	−3.5	0.00942353
XLOC_007772	Scin	12:40059768–40,134,228	5.36	0.5	−3.43	0.0391793
XLOC_012908	Krt6a	15:101689931–101,694,307	3.0	31.08	3.37	0.00942353
XLOC_022065	Iqgap3	3:88082044–88,121,048	1.09	10.98	3.33	0.00942353
XLOC_003382	–	10:79048734–79,059,175	1.67	0.17	−3.3	0.00942353
XLOC_002473	Chchd10	10:75935559–75,940,672	48.82	5.03	−3.28	0.00942353
XLOC_021250	–	2:148376283–148,382,190	1.74	0.19	−3.16	0.00942353
XLOC_028771	Fam71f2	6:29275646–29,290,927	8.97	1.06	−3.08	0.00942353
XLOC_000700	Cyb5r1	1:134405743–134,411,742	60.75	7.19	−3.08	0.00942353
XLOC_021249	–	2:148374139–148,376,129	1.33	0.16	−3.01	0.00942353
XLOC_007014	Rrm2	12:24708240–24,714,146	9.74	76.73	2.98	0.00942353
XLOC_004951	Cdc6	11:98907770–98,923,940	1.49	10.97	2.88	0.00942353
XLOC_011906	Grina	15:76246785–76,249,913	131.99	18.55	−2.83	0.00942353
XLOC_034952	Shcbp1	8:4735979–4,779,534	2.93	20.2	2.79	0.00942353
XLOC_032951	Fgf21	7:45613906–45,615,490	3.92	0.58	−2.76	0.00942353
XLOC_005606	Hmmr	11:40701394–40,733,422	3.37	22.21	2.72	0.00942353

Treated and untreated columns show group average FPKM values. FPKM is a unit of measurement of gene expression (fragments per kilobase of transcript per million mapped reads). Isoforms with the highest fold change between groups are shown at the top of the table. The fold change is the log2-fold change in FPKM between the treated and untreated groups. q-values shown are p-values that have been adjusted using the Benjamini-Hochberg false discovery rate (FDR) approach to correct for multiple testing. As a general guide, fold changes with q-values below 0.05 are considered significant

A novel transcript could also be the result of a previously unknown splicing event for a known gene or a previously unknown long noncoding RNA (Table 4).

**Table 4:** Novel isoforms. Table of the 20 most significantly differentially expressed novel isoforms

New Isoform ID	Closest Known Transcript	Locus	Treated FPKM	Untreated FPKM	Log^2^ Fold Change	q_value
XLOC_028771	Fam71f2	6:29275646–29,290,927	8.97	1.06	−3.08	0.00942353
XLOC_000700	Cyb5r1	1:134405743–134,411,742	60.75	7.19	−3.08	0.00942353
XLOC_011906	Grina	15:76246785–76,249,913	131.99	18.55	−2.83	0.00942353
XLOC_008363	Idi1	13:8885500–8,892,451	8.47	55.59	2.72	0.00942353
XLOC_034570	Mcm5	8:75109527–75,128,439	5.52	29.52	2.42	0.00942353
XLOC_004629	Coro6	11:77462410–77,470,484	8.76	1.76	−2.31	0.0478209
XLOC_013221	Tfrc	16:32608919–32,632,794	4.92	23.57	2.26	0.00942353
XLOC_033943	Mki67	7:135689785–135,716,379	1.74	8.18	2.24	0.00942353
XLOC_016993	Fads1	19:10182887–10,196,878	7.66	32.64	2.09	0.0227413
XLOC_010082	Nt5dc2	14:31131073–31,168,641	6.74	26.89	2.0	0.0391793
XLOC_014349	Srrm2	17:23803186–23,824,739	47.42	13.38	−1.83	0.00942353
XLOC_033943	Mki67	7:135689785–135,716,379	4.87	17.22	1.82	0.00942353
XLOC_015979	Lama3	18:12334023–12,584,803	25.18	7.54	−1.74	0.00942353
XLOC_018214	Nelf	2:25054354–25,062,889	20.9	6.28	−1.73	0.0343286
XLOC_011906	Grina	15:76246785–76,249,913	223.87	68.17	−1.72	0.00942353
XLOC_002731	Eea1	10:95940662–96,046,110	15.68	4.81	−1.7	0.0162365
XLOC_022994	Fdps	3:89093587–89,101,967	43.96	142.23	1.69	0.00942353
XLOC_027325	Kntc1	5:123749725–123,821,593	2.24	6.56	1.55	0.00942353
XLOC_023920	Smc2	4:52439242–52,488,364	4.49	13.04	1.54	0.00942353
XLOC_022503	Cenpe	3:135212562–135,273,540	6.55	18.92	1.53	0.0433531

Treated and untreated columns show group average FPKM values. FPKM is a unit of measurement of gene expression (fragments per kilobase of transcript per million mapped reads). Novel transcripts with the highest fold change between groups are shown at the top of the table. The fold change is the log2-fold change in FPKM between the treated and untreated groups. q-values shown are p-values that have been adjusted using the Benjamini-Hochberg false discovery rate (FDR) approach to correct for multiple testing. As a general guide, fold changes with q-values below 0.05 are considered significant

Novel transcripts now part of a regulatory network of Nkx2.1, miR-200c encapsulated and non-encapsulated with the direct targets of Nkx2.1 were classified with known features by listing the known transcripts most closely resembling the novel transcript with known feature and novel transcripts without any known features; given a locally unique name as the transcript identifier and a genomic positions for the features of the novel transcript, such as the location and number of exons. Differentially expressed novel isoforms also found are indicated in Tables 3 and 4.

### Mitochondrial membrane target gene expression

**Fig. 7:**
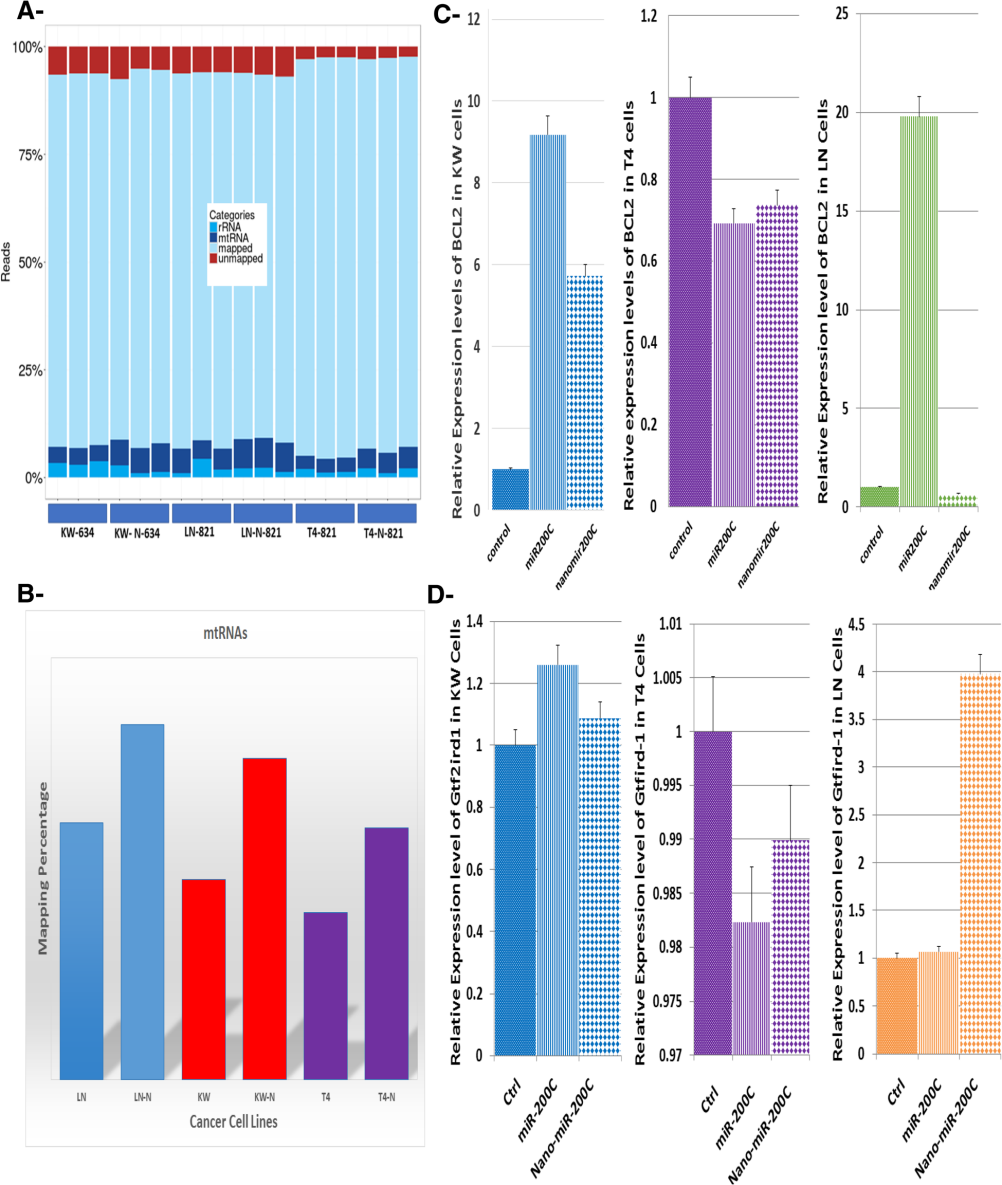
**a**- Summary of the mapping results for the reads by sample: KW-634, 821-LN, and 821-T4 untreated compared with treated cells. Each sample consists of reads that can be classified into the following categories: mapped (reads that align to the reference genome), outmapped or high abundance (e.g., rRNA, poly(A), poly(C), and mtRNA) and reads that do not align to anything (unmapped). **b**- The abundance of mtRNAs in cells treated with our Nano material compared with untreated cells in each cell line; there were no phenotypic effects or changes in the mitochondria but a significant reduction of cell growth. **c**- Endogenous BCL2 expression in mitochondria in mouse lung non-metastatic (KW-634, blue bars) and metastatic (821-T4, violet bars; 821-LN, green bars) cell lines, showing the various roles and effectiveness of miR-200c in each after treatment with 50 nM miR-200c and nano-miR-200c. The expression level of BCL2 was considerably elevated after treatment with miR-200c and Nano miR-200c in non-metastatic KW-634, reduced in metastatic 821-T4 and mixed in metastatic 821-LN. **d**- Similarly, Gtf2ird expression was slightly elevated in KW-634 (blue bars) treated with miR-200c/Nano miR-200c, decreased in 821-T4 (violet bars), and significantly elevated in 821-LN (orange bars)

Mapping of the sequencing data was a useful quality control steps in the NGS data analysis pipeline because it helped to evaluate the quality of the samples. Reads were classified into the following classes: 1- Mappable reads with alignment to the reference genome (including mRNA, pre-mRNA, poly (A) tail lncRNA and pri-miRNA); 2- outmapped reads or high abundance reads, such as rRNA, mtRNA, poly(A) and poly(C) homopolymers; 3- unmapped reads, indicating no alignment was possible. It was possible to align 83–93% of the reads to the reference genome, with little or no degraded rRNA material at approximately 1 to 4% and (Fig. 7a) summarize the mapping results with the total number of reads obtained for each sample. On average, 20.1 million reads were obtained for each sample, and the average genome-mapping rate was 94% (Fig. 7a). Outmapped or high abundance reads aligned for mtRNA showed a significant increase in mapping results for our Nano-miR-200c-treated samples compared with the controls (Fig. 7b). To clarify the significance of this result in metastatic and non-metastatic cells, we investigated the expression of mitochondrial genes BCL2 regulator of mitochondrial apoptosis (Assay ID: Mm00477631_m1 Lot No. 1550115 (Fig. 7c) and Gtf2ird (Assay ID: Mm01195467_m1 Lot No. P170228) (Fig. 7d) by qRT-PCR (Fig. 7). We generated a miR-200c-loaded nanoparticles below 100 nm in size that was able to affect mitochondrial gene regulation and expression within the mitochondria. This is a direct evidence of our formulation crossing the mitochondrial membrane.

### Nano miR-200c enhances or decreases migration and invasion while affecting protein signaling pathway activation

**Fig. 8:**
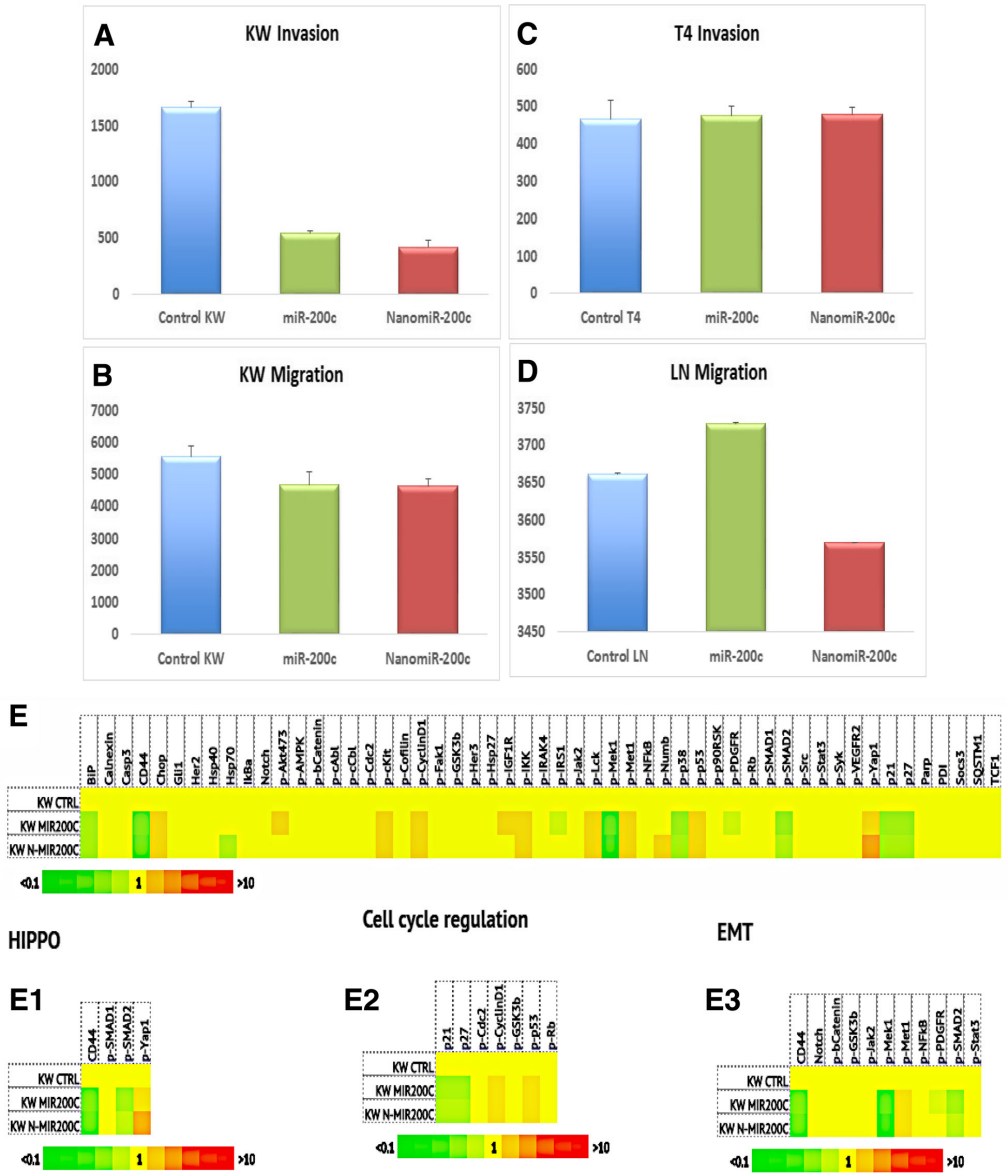
Invasion (**a**) and Migration (**b**) studies in mouse lung non-metastatic KW-634 cells; invasion assay in metastatic 821-T4 cells (**c**), and migration in metastatic 821-LN cells (**d**) (blue control, green miR-200c treatment, red Nano miR-200c treatment). Protein assay using the ActivSignal IPAD Platform to help understand the pathway activation/inhibition profiles of cells by assessing the signaling pathways in each sample of mouse lung non-metastatic KW-634 cells (**e**). The IPAD assay allows direct measurement of the expression and modification of components of signaling pathways. Hippo pathway activity (***e***_1_), Cell cycle regulation (***e***_2_) and EMT (***e***_3_)

Encapsulated miR-200c expression contributed to a significant decrease in migration, invasion and metastasis mechanism of mouse lung non-metastatic KW-634 (Fig. 8a and b) and metastatic 821-LN (Fig. 8d). Very little or no changes are observed in 821-T4 invasion (Fig. 8c) cells when compared using transwell assays with or without Matrigel. The invasion and migration ability of mouse lung non-metastatic (KW-634) cells transfected with Nano miR-200c were significantly decreased when compared cells treated with miR-200c alone and the controls (Fig. 8a and b). This can be due to the non-metastatic characteristic of the cells where both invasion and migration are affected. In contrast, by transfecting the cells with miR-200c or Nano miR-200c inhibitor into metastatic 821-T4 cells, invasion was not significantly impacted (Fig. 8d). The samples were processed and target proteins detected with content analysis to generate results using mouse lung non-metastatic KW-634 (Fig. 8e).

### ISH of lung development stages in CD-1, Kras and phosphorylation mutant lungs

**Fig. 9:**
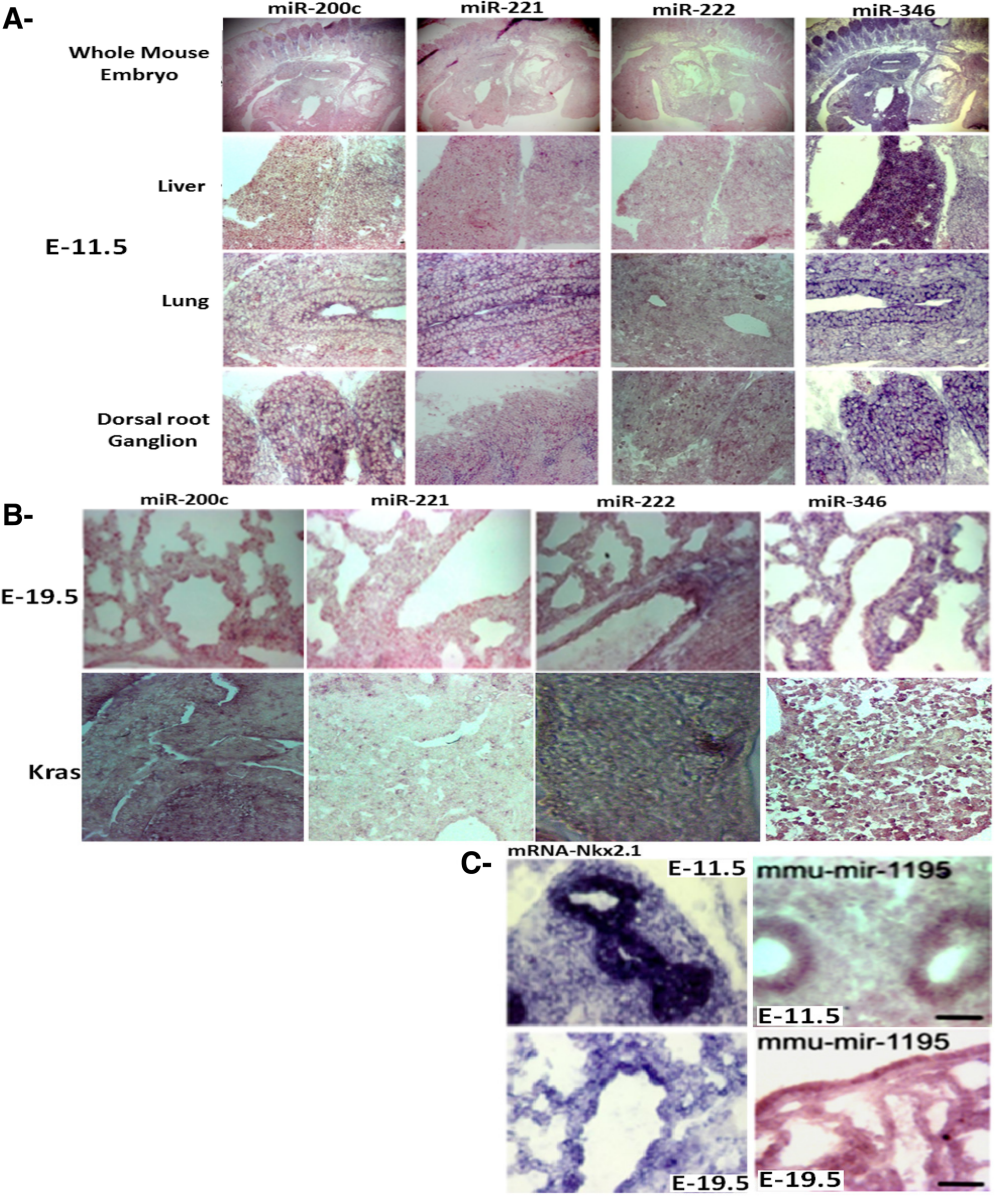
In situ hybridization analyses of the expression patterns of miRs- 200c, 221, 222, 346, and 1195 and Nkx2.1 in developmental CD1, phosphorylation mutant and Kras mice. **a**- Comparison of the levels of miR-200c, 221, 222, and 346 in our mouse models at E-11.5 in whole embryo, liver, lung, and dorsal root ganglion. miR-346, for instance, was expressed specifically in the liver and dorsal root ganglion. **b**- E-19.5 showed similar patterns of expression in normal mouse lung epithelium in comparison to Kras. **c**- In situ hybridization of Nkx2.1 mRNA at E11.5 confirmed the similar epithelial pattern to miR-1195

In situ hybridization (ISH) using lungs from early embryos (day-11 and day-19) and adults, revealed that miR-200c, miR-221, and miR-222 presented nonspecific patterns, while miRs 1195 and 346 had the same epithelial correlation as Nkx2.1; miR-200c was shown to be prominent in the dorsal root ganglion, while miR-346 was present in the liver and dorsal root ganglion (Fig. 9a). At E-19.5, their patterns were similar to adult Kras (Fig. 9b), and C- miR-1195 showed a similar epithelial pattern to both Nkx2.1 at E-11.5 and E-19.5 (Fig. 9c). miRs are shown here to be tissue and organ specific meaning we can specifically target the pathological process in the organ or used their potential miRs as a biomarkers of that particular disease.

## Discussion

Identifying Nkx2.1-dependent tumor-associated miRs and their target genes is critical for understanding the roles of miRs in tumorigenesis and is very important for novel therapeutic targets. From previous work, we have generated encapsulated drugs for cancer treatment and identified some relevant miRs. In this work, we focused on the delivery effect of encapsulated miR-200c on Kras non-metastatic KW-634 and metastatic 821-LN and 821-T4 and demonstrated an inhibition of invasion and migration with an upregulation of mitochondrial genes such as miR-200c, which plays the role of an epigenetic regulator in addition to its transcriptional regulatory role with a critical impact on downstream regulators. Our findings show that when encapsulated, miR-200c participated in the regulatory pathway of Nkx2.1 in lung cancer cells.

To our knowledge, this is the first study to examine the mechanism of encapsulated Nkx2.1-dependent miR-200c on lung cancer formation and metastasis. Both are the main cause of death in patients affected by this untreatable disease. To understand their functional implication in cancer with the possibility to translate them into the clinic, we have developed a Nano formulation with miR-200c as a treatment option. Nano formulations are attractive because the hydrophilic nature of miRs, their sensitivity to nuclease degradation, and inefficient uptake by tumor cells without the use of vectors are major problems with the introduction of miRs into tumor cells. Nano formulations protect the miR from degradation and do not have the toxicity and side effect profiles of viral delivery methods [39]. Our formulation is less than 100 nm and contains nontoxic and biodegradable particles, resulting in greater and extended cellular uptake and reducing the action of the mononuclear phagocyte system (MPS). Additionally, it is easily cleared from the body because of the biodegradable capabilities. Because of the dramatic decrease in particle size (Fig. 1a), surface-to-volume ratios of the Nano formulation containing miR-200c were increased, increasing the bioavailability of the cargo (Fig. 1b). The intracellular cargo release was efficiently induced by factors in the cell environment, such as pH, redox potential, and enzymes, allowing it to be used as a prolonged biodegradable protective shield with improvements in high target uptake and the efficiency of delivery of the payload. Our delivery methods protect miRs from nucleases and enhance uptake into tumor cells and block cancer cell movements, preventing them from invading surrounding tissue and increasing their bioavailability and efficacy. Interestingly, the formulations also enabled particle entrance into the mitochondrion, as evidenced by the high expression of miRs in the next-generation sequencing data.

Understanding the zeta potential variations of encapsulated Nano miR-200c is very important as this parameter controls the direction and magnitude of electro-osmotic permeability, with a crucial role in uptake and increasing the bioavailability and efficiency in the regulation of target genes. Zeta potential is evaluated by measuring the electrophoretic or electro osmotic mobility [40] or by measuring the generated streaming current or streaming potential (Fig. 1c). Our zeta potential was slightly negative as a positive charge induces toxicity [41], directly affecting the performance and processing characteristics of our delivery while increasing the efficacy.

This capacity to enter cells has been attributed to the small diameter of Nanoparticles, allowing greater penetrance through the lipid bilayer. Our Nanoparticle shows great potential for guiding drugs within the cell. We were able to observe mitochondria-RFP behavior in cells with more than 90% efficiency using a fluorescent imaging system and standard TRITC/RFP filter set. We studied the staining of mitochondria, which does not depend on mitochondrial membrane potential. In addition, we also co-transduced and transfected more than one CellLight® reagent, labeling live cells with multiple tracking and tracing dyes to image dynamic cellular processes. For drug and gene therapy, the deliveries must be to the nucleus, wherein genetic variables play a major role [42].

### Cell growth and uptake, Nkx2.1, miR-200c targets, RT-qPCR

Conventional chemotherapy disrupts the functions of cell organelles, such as the mitochondria, and our Nano formulations acted in a similar manner. The mitochondria are involved in many cellular processes that could be disrupted by our Nano formulation, such as dynamic fusion, division, morphology and, most importantly, apoptosis. Thus, a mitochondrial membrane assay was performed to ascertain whether our Nano formulation could affect mitochondrial dynamics. After treatment, staining of the mitochondrial membrane was carried out and changes to key mitochondrial factors were observed. RT-qPCR was performed to analyze the expression level of genes with expression related to miR-200c as many miRNAs are transcriptionally regulated by key signaling pathways such as Hippo which is responsive to, and regulates, many cellular properties that have been linked to tumorigenesis, Yap/Taz-p degradation in the cytoplasm and the balance of apoptosis and cell proliferation in the nucleus; cell cycle regulation, and EMT; all are important mediators of signal transduction pathways. miR-200c silenced signal transduction pathways by targeting kinases or potentiated signaling events by regulating important enzymes. Thus, we have developed a naturally evolving strategy for miR-200c to overcome the mitochondria to target the nuclear envelope and enter the nucleus, which can be utilized, optimized and integrated to establish an efficient delivery system.

### Next-generation sequencing (NGS)

We were able to establish the regulatory relationship between Nkx2.1 and miR-200c in particular, as well as their direct target genes. While comparing the outcomes of treatment of our cells with Nano miR-200c and simple miR-200c, we identified higher fold changes in expression of Nkx2.1, Myb, Six4 and Six1. The therapeutic delivery of Nano miR-200c increased the expression level of miR-29b, a regulator of EMT (Additional file 2: Table S1), and miR-1247, an inhibitor of invasion and inducer of apoptosis (Additional file 2: Tables S1 and S2). Nkx2.1 and miR-200c act as suppressors of tumor growth and metastasis as part of the network presenting an option for lung cancer treatment. We observed a sensitive reduction in the growth of our cancer cells lines after 24 h of treatment.

Our study showed pronounced biological and treatment differences between the samples, describing the primary components of the variation in our data. This led to the separation of samples in different regions of a PCA plot corresponding to their biology and treatments. Fewer variable factors and reduced variability of cell quality were observed, which ensured that the samples clustered according to biology and treatments. During the transcriptome assembly process, both known and novel transcripts were identified.

### The mitochondrial membrane target gene expression

mtRNA showed a significant increase in mapping results for our Nano miR-200c-treated samples compared with the controls, and this interesting finding confirmed the mitochondrial enhancement of our preparation. Mitochondria participate in many cellular and physiologic processes, and we believe our formulations enhance morphological changes in mitochondria, providing new mechanisms and insights into the dynamics of our miR-200c. However, there were no phenotypic changes in our mitochondria in live cells based on staining with red fluorescent protein (RFP), implying that miR-200c enters the mitochondria but does not affect their phenotype but their gene expression.

### Cancer cell migration, invasion and protein

We found that miR-200c in our Nano vehicle governed cell migration and invasion more efficiently via a complex network of signal transduction pathways involving Hippo, cell cycle regulation, and EMT, compared to non-encapsulated miR-200c. miR-200c affected the oncogenic properties of non-metastatic and metastatic lung tumor cells by regulating cell cycle progression, migration, invasion, and tumor growth. The pharmacological Nano miR-200c compound impaired invasive tumor cell behavior in different migration and invasion models in vitro. By significantly affecting the invasion and migration mechanisms important for cell dissemination and the expression program of lung cancer cells, and by contributing to the inhibition of Kras, EMT, Hippo and cell cycle regulation pathways, thus functioning as metastasis blockers, technologies such as our Nano miR-200c formulation could provide potential therapeutic and/or prognostic targets. Nano formulations increase the regulation of cells by targeting cancer cells specifically, thus impacting tumor formation and progression. For instance, within the Hippo signaling pathway, which allows cellular control of organ size and tumor suppression, Yap1 could be targeted in the cytoplasm in cells treated with Nano miR-200c, leading to cell degradation. In contrast, since our formulation allows nuclear miR-200c, it could possibly induce apoptosis in the nucleus. This phenomenon can also be observed with high levels of the cell cycle regulatory components P-53 and p-IKK characterizing DNA damage**.** miR-200c in an encapsulated molecule governed cell migration and invasion via a complex network of signal transduction pathways that involve Hippo, cell cycle regulation, and EMT, among others.

### In situ hybridization (ISH)

Using ISH approaches, we attempted to identify and characterize miRNA expression patterns of Nkx2.1, miR-200c, miR-221, miR-222, miR-346, and miR-1195 in the developing lungs of CD1, Nkx2.1 phosphorylation mutant and Kras mice. Although the low abundances of long miRNA precursors and short nucleotide sequences of mature miRNAs in animals have hampered analysis of their expression patterns by ISH, we were able to successfully identify miR-1195 in the epithelium. Lungs were isolated at multiple time points from normal CD1 and mutant mice, and the expression of Nkx2.1 and numerous miRs were assessed in E11.5 and E19.5 embryos first by confocal microscopy analyses, which showed that Nkx2.1 and some miRs such as miR-200c and miR-1195, a marker of cell proliferation [43], were co-localized in most epithelial nuclei in early lung development (E11.5) and in late development (E19.5). However, they were expressed only in a few cells in the distal lung, and those cells did not express Nkx2.1 protein, as shown previously. miR-200c and miR-1195 were detected by in situ hybridization in developing mouse lungs, and their expression levels were altered in lungs lacking functional phosphorylated Nkx2–1. Moreover, NKX2.1 protein was bound to regulatory regions of these miRs. By in situ hybridization, we determined that miR-1195; an Nkx2.1 variant was expressed in normal developing lung epithelium in a similar pattern to Nkx2.1, suggesting a possible role for miR-1195 as a mediator of Nkx2.1 inhibitory or silencing function. As an intracellular marker, miR-200c was found to accumulate in epithelial buds in developing lungs and was negatively regulated by the epithelial marker Nkx2.1.

The expression of Nkx2.1 in the anterior foregut was previously detected by ISH in the thyroid field of the anterior foregut at E8.5 [44]. The appearance of Nkx2.1 transcripts at E9.0 in the prospective lung field allowed for identification of the first lung epithelial progenitor cells. Nkx2.1 was expressed in the primary lung buds and subsequently in the branching epithelium, with no detectable expression in the lung mesenchyme. Later in development, intense Nkx2.1 staining could be seen in both the alveolar epithelium and conducting airways [45–48]. Whole mount ISH analysis revealed the same defined patterns of Nkx2.1 and miR-1195 expression in the embryos at E11.5 and E19.5. These findings support the hypothesis that cells expressing Nkx2.1 at early and late stages of lung development are in different stages of the cell cycle and undergoing different biological processes, which are likely determined by differential patterns of gene expression.

## Conclusions

The therapeutic delivery of miR-200c suppresses lung cancer and prevents metastasis in culture, positioning it as a potential therapeutic agent. Many miRs prepared for delivery on tumor cells have been developed, such as lentiviruses, adenovirus vectors, expression vectors, transfections, and synthetic miR precursors, but they are not completely efficient due to their toxicity and non-specificity [49–51]. Many miRs are also used as co-adjuvants, such as miR-21 in combination with topotecan and Taxol for breast cancer [52, 53]. We modified cancer cell invasion and migration mechanisms, the main causes of mortality in cancer patients, simply by delivering our encapsulated target miR-200c with improved stability and delivery and thus overcoming the poor penetration of tumor tissues and poor intracellular delivery, which have been insurmountable challenges. The biodegradability of the encapsulation reduced off-target effects, and the packaging allowed enhanced bioavailability with sufficient miR processing enzymes. This is the first evidence to show that encapsulated miR-200c is part of a complex cytoplasmic, nuclear and mitochondrial network of pathways that involve lipids, enzymes, and proteins with new mechanisms and potential therapeutic targets for lung cancer. In conclusion, our delivery system for miR-200c may provide a more efficacious treatment for lung cancer with fewer adverse side effects given our novel data, indicating that cell mitochondria are not directly damaged phenotypically but are regulated genetically.

## Additional files

Additional file 1: **Figure S1.** Stable dynamic laser light scattering particle size analysis of Nano miR-200c with the Z-average size distribution of the particle microfluidization resulted in a dramatic decrease in particle size and the demonstration of the particle size heterogeneity, even within what appeared to be a homogeneous distribution 6 months later. **Figure S2.** Cell cycle FACS analysis of various encapsulated miRs (200c, 221, 222, 346, and 1195) with negative empty vector control (same composition as the suspension or vehicle with no miRs). **Figure S3.** A- Heat map and unsupervised hierarchical clustering by sample and genes were performed for the listed samples using the 500 genes with the largest coefficient of variation based on the FPKM counts. B- Scatter plot for significantly enriched GO terms associated with genes that were differentially expressed between the treated and untreated cells; The plot shows a comparison of the results obtained with the two statistical tests used. Values along the diagonal line were consistent between both methods. Values on the bottom left of the plot correspond to the terms with most reliable estimates using both methods. The size of the dot is proportional to the number of genes mapping to that GO term, and the coloring represents the number of significantly differentially expressed transcripts corresponding to the term, with dark red representing more terms and yellow fewer terms. C- Volcano plot showing the relationship between the *p*-values and the log2-fold change in normalized expression (FPKM) between treated and untreated cells; Data are based on the normalized FPKM (abundance) for each gene per sample. Data are based on treated Vs untreated groups of non-metastatic KW-634 metastatic 821-LN, and 821 T4. **Figure S4.** NGS comparison of untreated (A- KW/LN, B-LN/T4, and C- KW/T4) and treated (D- KW/LN and E-LN/T4) groups’ shows a complete reversal of gene expression. F- KW-634/821-T4: Heat map and unsupervised hierarchical clustering by sample and genes were performed for the listed samples using the 500 genes with the largest coefficient of variation based on FPKM counts. Data are based on samples from A- KW_untreated and LN_untreated, B- LN_untreated and T4_untreated, C- KW_untreated and T4_untreated, D- KW_treated and LN_treated, E- LN_treated and T4_treated, F- KW_treated and T4_treated groups, based on the normalized FPKM (abundance) for each gene per sample (raw data for the genes), and G- all the possible combinations clustering based on Pearson Correlation. It is very interesting that B and E genes showed completely reversed expression when treated cells were compared with each other. (DOCX 1732 kb)

Additional file 2: **Table S1.** Genes: Table of the 20 most significantly differentially expressed genes. KW_treated and KW_untreated columns show group average FPKM values. FPKM is a unit of measurement of gene expression (fragments per kilobase of transcript per million mapped reads). Transcripts with the highest fold change between groups are shown at the top of the table. The fold change is the log2-fold change in FPKM between the KW_treated and KW_untreated groups. q-values shown are *p*-values that have been adjusted using the Benjamini-Hochberg false discovery rate (FDR) approach to correct for multiple testing. As a general guide, fold changes with q-values below 0.05 are considered significant. **Table S2.** Genes: Table of the 20 most significantly differentially expressed genes. LN_treated and LN_untreated columns show group average FPKM values. FPKM is a unit of measurement of gene expression (fragments per kilobase of transcript per million mapped reads). Transcripts with the highest fold change between groups are shown at the top of the table. The fold change is the log2-fold change in FPKM between the LN_treated and LN_untreated groups. q-values shown are p-values that have been adjusted using the Benjamini-Hochberg false discovery rate (FDR) approach to correct for multiple testing. As a general guide, fold changes with q-values below 0.05 are considered significant. **Table S3.** Genes: Table of the 20 most significantly differentially expressed genes. T4_treated and T4_untreated columns show group average FPKM values. FPKM is a unit of measurement of gene expression (fragments per kilobase of transcript per million mapped reads). Transcripts with the highest fold change between groups are shown at the top of the table. The fold change is the log2-fold change in FPKM between the T4_treated and T4_untreated groups. q-values shown are *p*-values that have been adjusted using the Benjamini-Hochberg false discovery rate (FDR) approach to correct for multiple testing. As a general guide, fold changes with q-values below 0.05 are considered significant. (DOCX 18 kb)
