# Supplementary figures and images for: Encapsulated miR-200c and Nkx2.1 in a nuclear/mitochondria transcriptional regulatory network of non-metastatic and metastatic lung cancer cells
Source: BMC Cancer. 2019 Feb 11;19:136. doi: 10.1186/s12885-019-5337-6 (PMC6371494; doi:10.1186/s12885-019-5337-6)

**Supplemental (S) Figures (Fig):**

Fig S1:


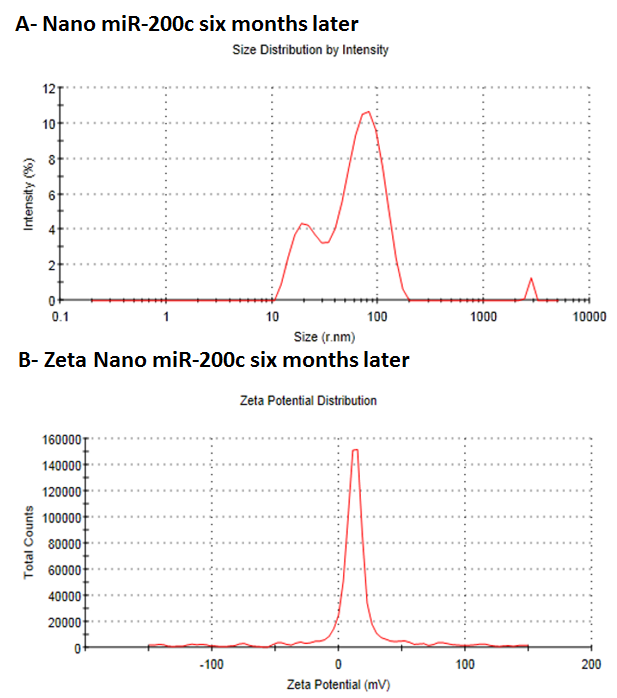


Fig S2:


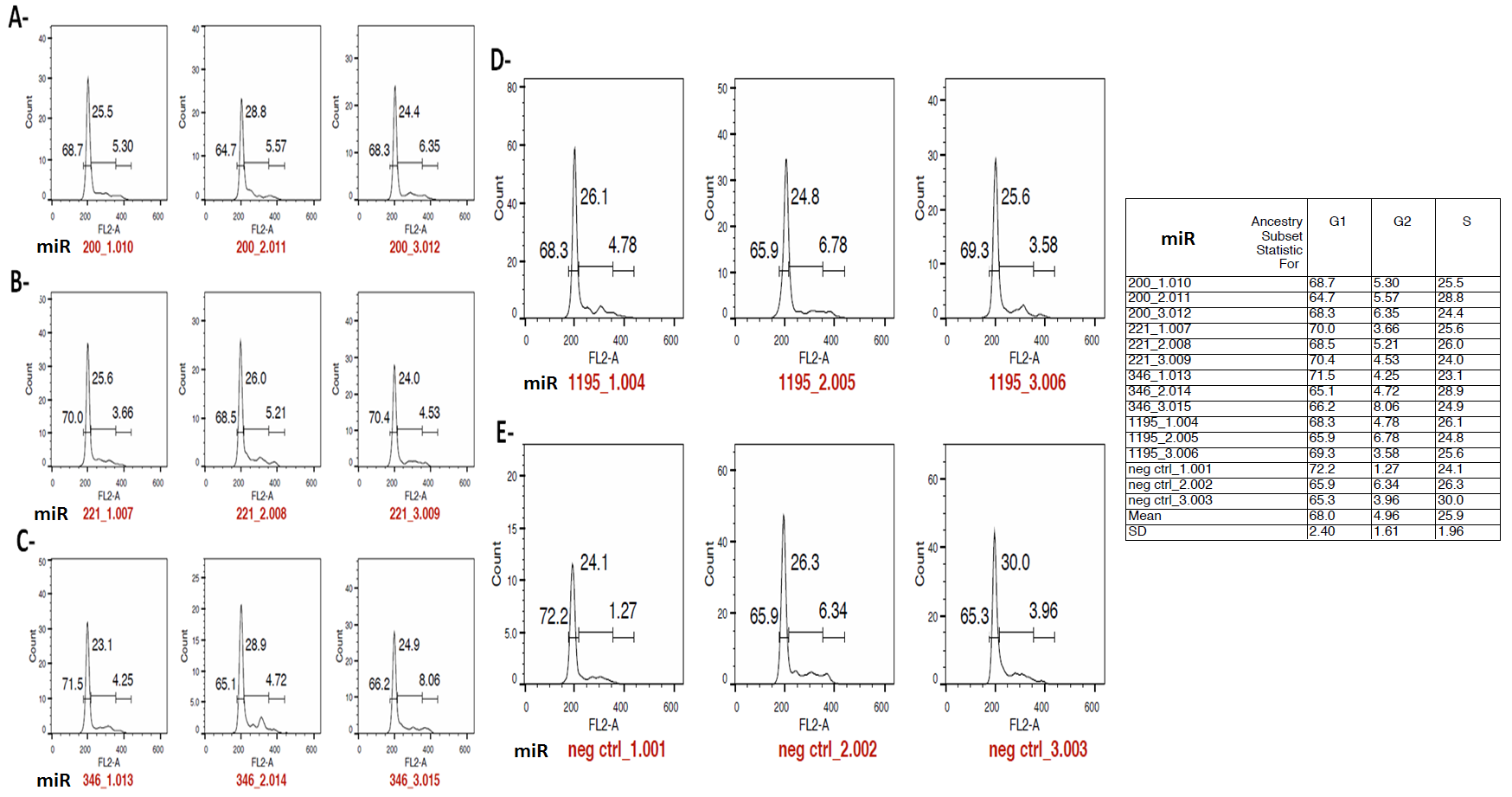


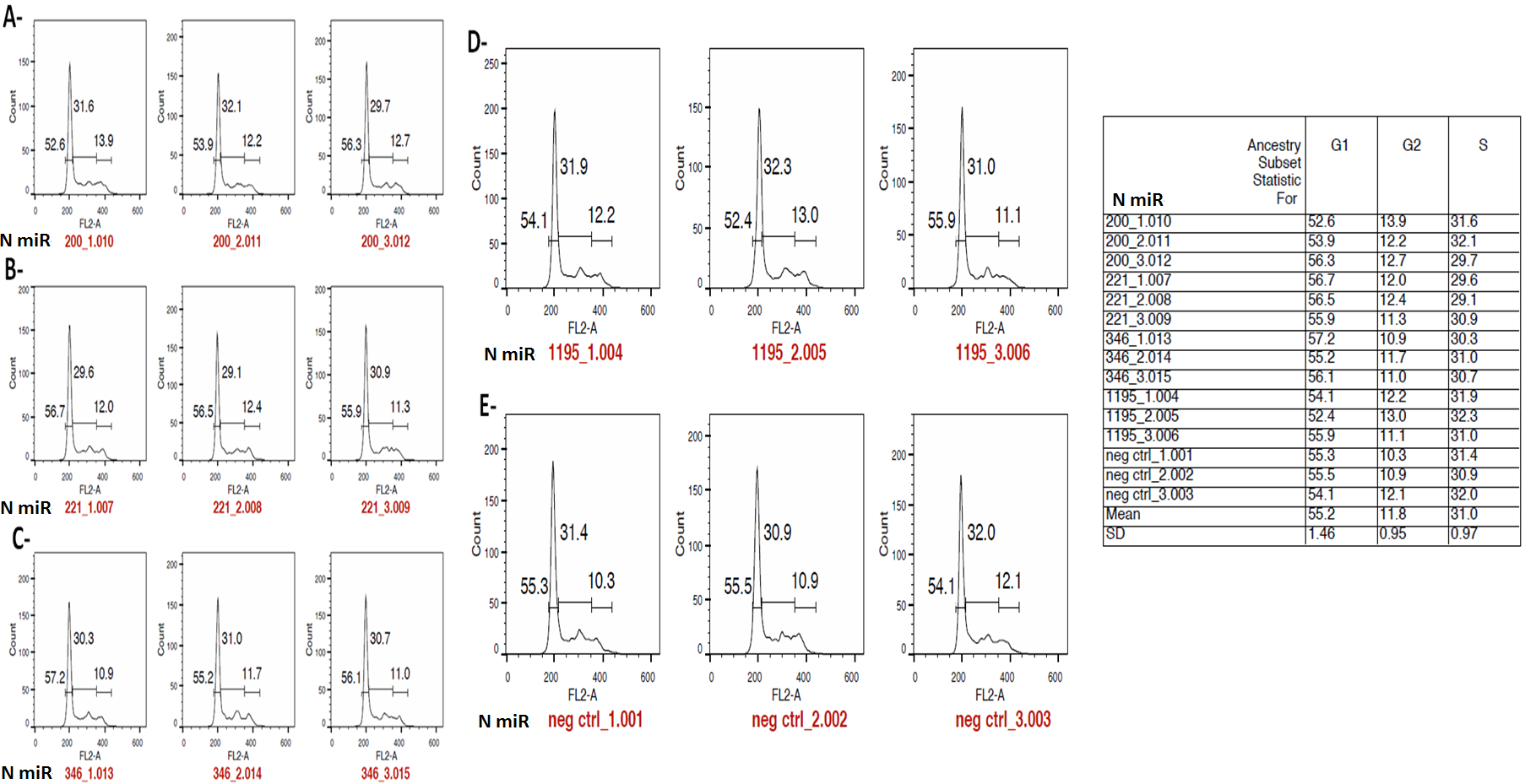


Fig S3:

**
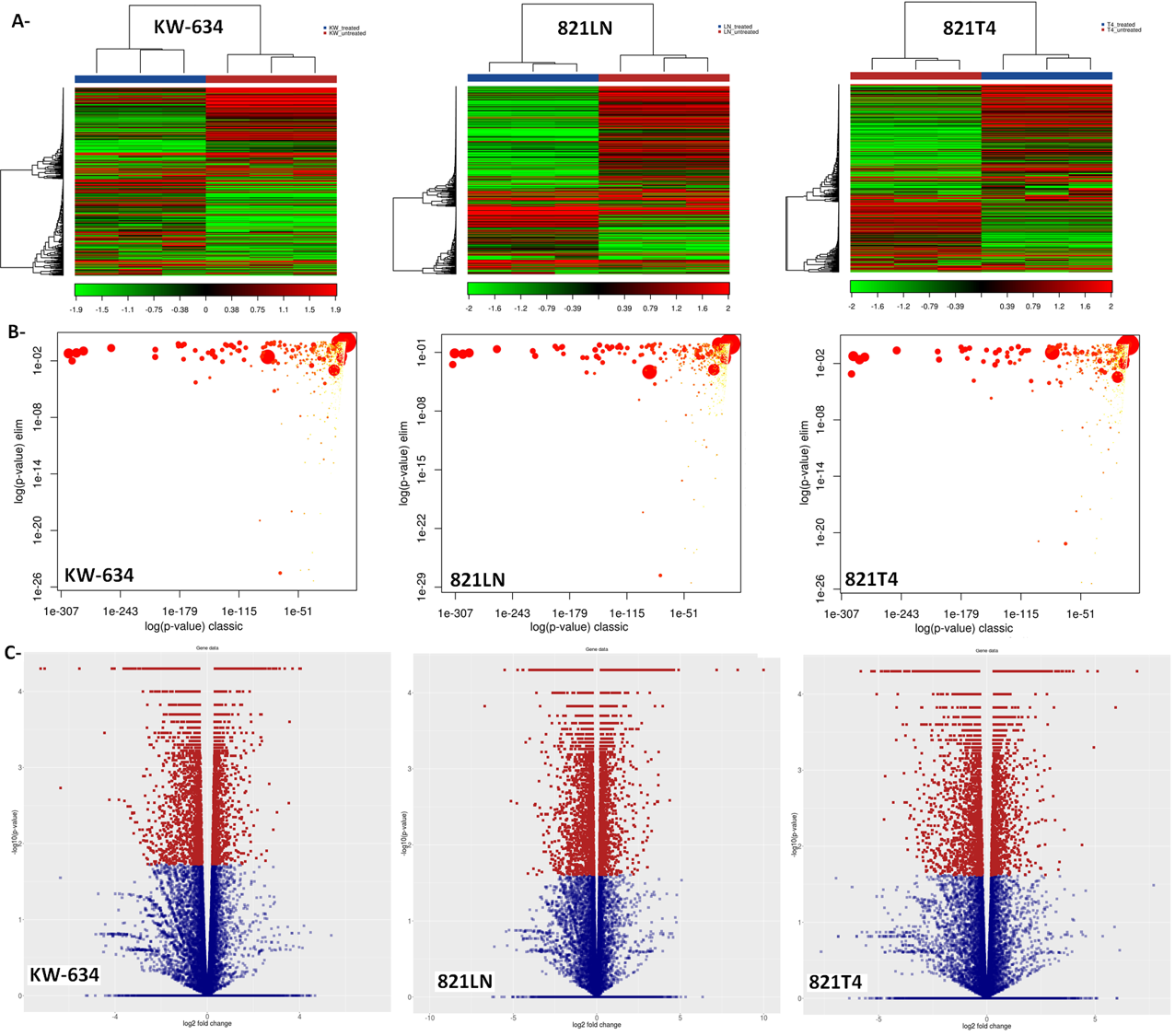
**

Fig S4:


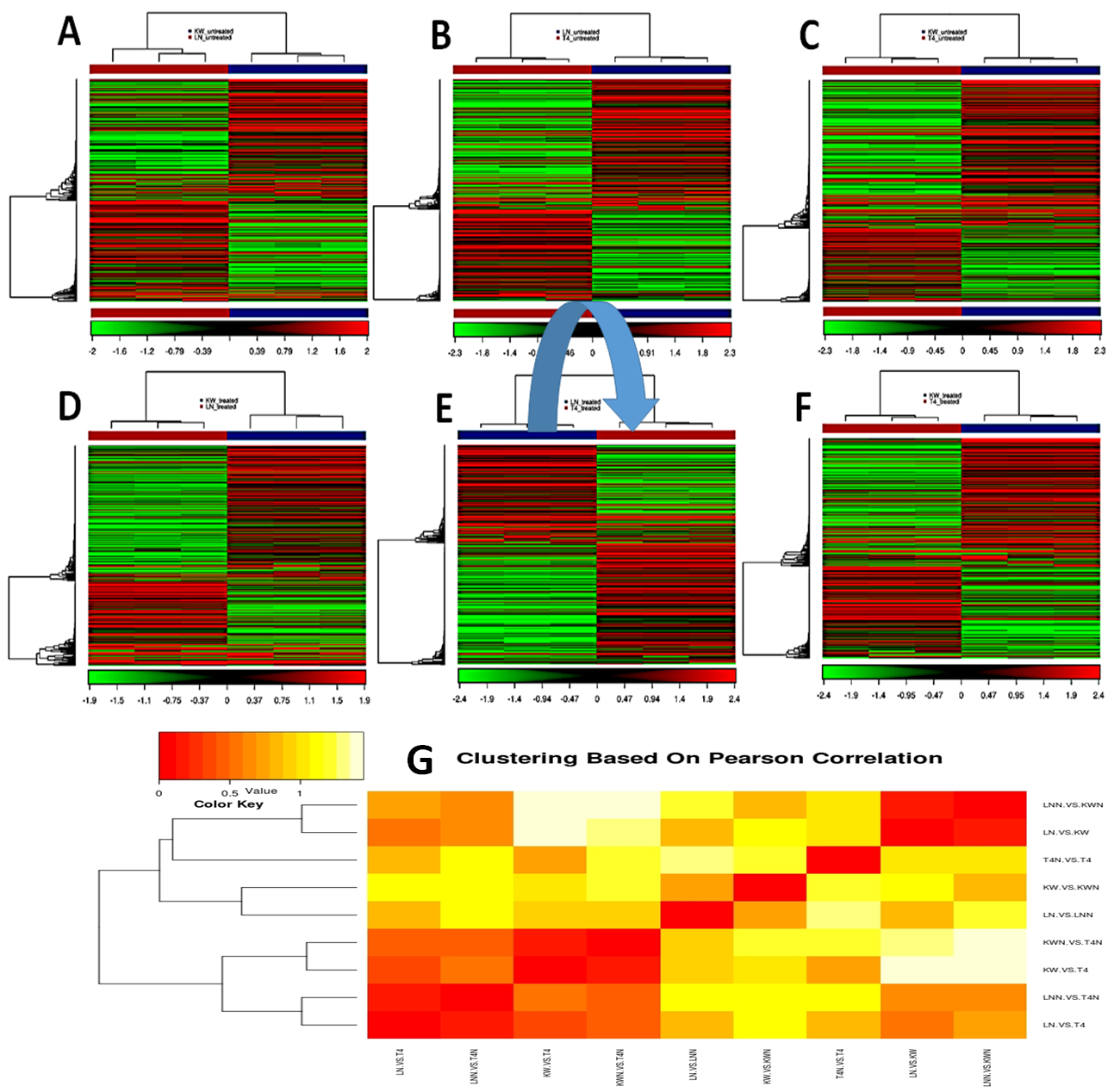

Supplement: Supplementary file 1 — Figure S1. Stable dynamic laser light scattering particle size analysis of Nano miR-200c with the Z-average size distribution of the particle microfluidization resulted in a dramatic decrease in particle size and the demonstration of the particle size heterogeneity, even within what appeared to be a homogeneous distribution 6 months later. Figure S2. Cell cycle FACS analysis of various encapsulated miRs (200c, 221, 222, 346, and 1195) with negative empty vector control (same composition as the suspension or vehicle with no miRs). Figure S3. A- Heat map and unsupervised hierarchical clustering by sample and genes were performed for the listed samples using the 500 genes with the largest coefficient of variation based on the FPKM counts. B- Scatter plot for significantly enriched GO terms associated with genes that were differentially expressed between the treated and untreated cells; The plot shows a comparison of the results obtained with the two statistical tests used. Values along the diagonal line were consistent between both methods. Values on the bottom left of the plot correspond to the terms with most reliable estimates using both methods. The size of the dot is proportional to the number of genes mapping to that GO term, and the coloring represents the number of significantly differentially expressed transcripts corresponding to the term, with dark red representing more terms and yellow fewer terms. C- Volcano plot showing the relationship between the p-values and the log2-fold change in normalized expression (FPKM) between treated and untreated cells; Data are based on the normalized FPKM (abundance) for each gene per sample. Data are based on treated Vs untreated groups of non-metastatic KW-634 metastatic 821-LN, and 821 T4. Figure S4. NGS comparison of untreated (A- KW/LN, B-LN/T4, and C- KW/T4) and treated (D- KW/LN and E-LN/T4) groups’ shows a complete reversal of gene expression. F- KW-634/821-T4: Heat map and unsupervised hierarchical clustering by sa [file 12885_2019_5337_MOESM1_ESM.docx]
