# Supplementary material for: Encapsulated miR-200c and Nkx2.1 in a nuclear/mitochondria transcriptional regulatory network of non-metastatic and metastatic lung cancer cells
Source: BMC Cancer. 2019 Feb 11;19:136. doi: 10.1186/s12885-019-5337-6 (PMC6371494; doi:10.1186/s12885-019-5337-6)
Supplement: Supplementary file 2 — Table S1. Genes: Table of the 20 most significantly differentially expressed genes. KW_treated and KW_untreated columns show group average FPKM values. FPKM is a unit of measurement of gene expression (fragments per kilobase of transcript per million mapped reads). Transcripts with the highest fold change between groups are shown at the top of the table. The fold change is the log2-fold change in FPKM between the KW_treated and KW_untreated groups. q-values shown are p-values that have been adjusted using the Benjamini-Hochberg false discovery rate (FDR) approach to correct for multiple testing. As a general guide, fold changes with q-values below 0.05 are considered significant. Table S2. Genes: Table of the 20 most significantly differentially expressed genes. LN_treated and LN_untreated columns show group average FPKM values. FPKM is a unit of measurement of gene expression (fragments per kilobase of transcript per million mapped reads). Transcripts with the highest fold change between groups are shown at the top of the table. The fold change is the log2-fold change in FPKM between the LN_treated and LN_untreated groups. q-values shown are p-values that have been adjusted using the Benjamini-Hochberg false discovery rate (FDR) approach to correct for multiple testing. As a general guide, fold changes with q-values below 0.05 are considered significant. Table S3. Genes: Table of the 20 most significantly differentially expressed genes. T4_treated and T4_untreated columns show group average FPKM values. FPKM is a unit of measurement of gene expression (fragments per kilobase of transcript per million mapped reads). Transcripts with the highest fold change between groups are shown at the top of the table. The fold change is the log2-fold change in FPKM between the T4_treated and T4_untreated groups. q-values shown are p-values that have been adjusted using the Benjamini-Hochberg false discovery rate (FDR) approach to correct for multiple testing. As a general guide, [file 12885_2019_5337_MOESM2_ESM.docx]

**Supplemental (S) Tables:**

Table S1:

| **Gene ID** | **Gene** | **Locus** | **KW_treated**  **FPKM** | **KW_untreated FPKM** | **Log^2^Fold**  **change** | **q_value** |
| --- | --- | --- | --- | --- | --- | --- |
| **XLOC_001070** | **Mir29b2,Mir29c,**  **mmu-mir-29b-2** | 1:194938820-195037908 | 1262.69 | 0.24 | -12.39 | 0.000291648 |
| **XLOC_008145** | **AL591207.1,**  **Mir1247** | 12:110275383-110278963 | 1784.09 | 11.86 | -7.23 | 0.000291648 |
| **XLOC_030815** | **Cacng8** | 7:3394116-3415605 | 85.99 | 0.65 | -7.05 | 0.000291648 |
| **XLOC_026299** | **Clcnkb** | 4:141398828-141416014 | 0.88 | 0.01 | -6.36 | 0.00722027 |
| **XLOC_011996** | **Tnrc6b** | 5:80711318-80941086 | 176.72 | 3.79 | -5.54 | 0.000291648 |
| **XLOC_036671** | **Vipr1** | 9:121642715-121672954 | 1.33 | 0.06 | -4.46 | 0.00170005 |
| **XLOC_019817** | **-** | 2:177488270-177490941 | 1.47 | 0.08 | -4.25 | 0.00980537 |
| **XLOC_014247** | **Ncrna00085** | 17:17830342-17843009 | 419.65 | 24.01 | -4.13 | 0.000291648 |
| **XLOC_023519** | **Rabggtb** | 3:153907285-153912966 | 1815.33 | 104.78 | -4.11 | 0.000291648 |
| **XLOC_032977** | **Saa3** | 7:46711997-46715676 | 1.77 | 29.3 | 4.05 | 0.000291648 |
| **XLOC_007818** | **Ralgapa1** | 12:55602904-55821580 | 85.93 | 5.27 | -4.03 | 0.000291648 |
| **XLOC_035441** | **Mast1** | 8:84908559-84937359 | 2.85 | 45.83 | 4.01 | 0.000291648 |
| **XLOC_003469** | **-** | 10:81895524-81896098 | 2.83 | 0.2 | -3.84 | 0.00980537 |
| **XLOC_038048** | **Lonrf3** | X:36328352-36366853 | 0.93 | 12.05 | 3.69 | 0.000291648 |
| **XLOC_003379** | **-** | 10:79038548-79046643 | 5.02 | 0.41 | -3.63 | 0.000291648 |
| **XLOC_034056** | **-** | 7:145205866-145206552 | 1.8 | 0.15 | -3.59 | 0.0101216 |
| **XLOC_022259** | **Hist2h2bb** | 3:96267074-96279001 | 0.4 | 4.68 | 3.56 | 0.00126252 |
| **XLOC_000341** | **U2** | 1:72226239-72226430 | 3.47 | 40.3 | 3.54 | 0.0107418 |
| **XLOC_008396** | **-** | 13:14677687-14682438 | 0.98 | 0.09 | -3.52 | 0.000291648 |
| **XLOC_037598** | **-** | 9:124149098-124153080 | 1.63 | 0.14 | -3.49 | 0.000291648 |

Table S2:

| **Gene ID** | **Gene** | **Locus** | **LN_treated**  **FPKM** | **LN_untreated FPKM** | **Log^2^ Fold**  **change** | **q_value** |
| --- | --- | --- | --- | --- | --- | --- |
| **XLOC_034022** | **H19, Mir675** | 7:142575528-142578143 | 0.34 | 350.03 | 9.99 | 0.000180366 |
| **XLOC_008145** | **AL591207.1,**  **Mir1247** | 12:110275383-110278963 | 2.3 | 800.02 | 8.44 | 0.000180366 |
| **XLOC_011996** | **Tnrc6b** | 15:80711318-80941086 | 4.42 | 642.26 | 7.18 | 0.000180366 |
| **XLOC_032494** | **Gpr77** | 7:16234584-16244154 | 7.53 | 0.07 | -6.7 | 0.000504212 |
| **XLOC_013580** | **AC115005.1,**  **Gm7541** | 16:8622332-8624782 | 19.78 | 0.43 | -5.51 | 0.000180366 |
| **XLOC_003693** | **-** | 10:122058743-122060498 | 1.6 | 0.05 | -5.15 | 0.007017 |
| **XLOC_002067** | **Cenpf** | 1:189640535-189688086 | 1.14 | 34.09 | 4.9 | 0.000180366 |
| **XLOC_001102** | **Snord87** | 1:9908637-9944287 | 2520.29 | 91.33 | -4.79 | 0.00749846 |
| **XLOC_026432** | **Car6** | 4:150187014-150201332 | 4.64 | 0.17 | -4.74 | 0.000180366 |
| **XLOC_034523** | **Ankle1** | 8:71406009-71410532 | 0.37 | 9.07 | 4.62 | 0.000180366 |
| **XLOC_036264** | **2810417H13Rik** | 9:65828929-65908794 | 2.48 | 55.05 | 4.47 | 0.000180366 |
| **XLOC_002346** | **Gp49a,Lilrb4** | 10:51480631-51496613 | 54.27 | 2.54 | -4.42 | 0.000180366 |
| **XLOC_011416** | **Esco2** | 14:65818411-65833994 | 0.45 | 9.37 | 4.39 | 0.000180366 |
| **XLOC_028121** | **A430089I19Rik** | 5:94302518-94306796 | 0.04 | 0.92 | 4.38 | 0.00689508 |
| **XLOC_033943** | **Mki67** | 7:135689785-135716379 | 3.3 | 63.43 | 4.26 | 0.000180366 |
| **XLOC_028442** | **5930412G12Rik** | 5:128579105-128600693 | 0.92 | 0.05 | -4.25 | 0.00140176 |
| **XLOC_023082** | **Gm10696** | 3:94174411-94178193 | 0.07 | 1.27 | 4.24 | 0.000180366 |
| **XLOC_035219** | **Neil3** | 8:53586866-53639065 | 0.46 | 8.64 | 4.22 | 0.000180366 |
| **XLOC_022616** | **Depdc1a** | 3:159495432-159530059 | 0.77 | 13.64 | 4.14 | 0.000180366 |
| **XLOC_002625** | **-** | 10:82115069-82116575 | 1.15 | 0.07 | -4.12 | 0.0485902 |

Table S3:

| **Gene ID** | **Gene** | **Locus** | **T4_treated**  **FPKM** | **T4_untreated FPKM** | **Log^2^ Fold**  **change** | **q_value** |
| --- | --- | --- | --- | --- | --- | --- |
| **XLOC_036560** | **Celsr3** | 9:108826319-108852963 | 0.13 | 15.38 | 6.93 | 0.000180339 |
| **XLOC_030815** | **Cacng8** | 7:3394116-3415605 | 0.27 | 16.69 | 5.95 | 0.000511727 |
| **XLOC_002346** | **Gp49a,Lilrb4** | 10:51480631-51496613 | 18.72 | 0.33 | -5.83 | 0.000180339 |
| **XLOC_036797** | **Angptl6** | 9:20868641-20879727 | 45.29 | 1.13 | -5.32 | 0.000180339 |
| **XLOC_015300** | **-** | 17:27545755-27547350 | 5.41 | 0.14 | -5.29 | 0.000180339 |
| **XLOC_024975** | **-** | 4:150736119-150739106 | 3.05 | 0.08 | -5.2 | 0.000180339 |
| **XLOC_037321** | **AC163666.1,**  **SNORD50** | 9:88595218-88599516 | 1.16 | 39.98 | 5.11 | 0.000180339 |
| **XLOC_023439** | **AC123608.1,**  **Mir1895** | 3:133955472-134262156 | 0.92 | 31.64 | 5.11 | 0.000180339 |
| **XLOC_026432** | **Car6** | 4:150187014-150201332 | 4.87 | 0.14 | -5.09 | 0.000348917 |
| **XLOC_009329** | **Prl7a1** | 13:27633379-27642474 | 0.1 | 3.18 | 4.94 | 0.00154927 |
| **XLOC_024675** | **Ubxn11** | 4:134102569-134128789 | 5.9 | 0.2 | -4.88 | 0.000180339 |
| **XLOC_019290** | **Ebf4** | 2:130295168-130370685 | 3.5 | 0.12 | -4.84 | 0.000180339 |
| **XLOC_012110** | **C230037L18Rik** | 15:89472475-89484847 | 2.93 | 0.11 | -4.74 | 0.0113744 |
| **XLOC_012225** | **Soat2** | 15:102150517-102163474 | 9.47 | 0.36 | -4.71 | 0.000180339 |
| **XLOC_018145** | **BC061194,**  **Gm13334** | 2:18694031-18760862 | 0.81 | 0.03 | -4.7 | 0.00347889 |
| **XLOC_012908** | **Krt6a** | 15:101689931-101694307 | 2.52 | 63.45 | 4.66 | 0.000180339 |
| **XLOC_011152** | **4930431P03Rik** | 14:44851234-45006900 | 0.83 | 0.03 | -4.63 | 0.000180339 |
| **XLOC_019866** | **Col20a1** | 2:180985800-181017540 | 11.17 | 0.48 | -4.54 | 0.000180339 |
| **XLOC_030586** | **-** | 6:128169704-128183511 | 2.86 | 0.13 | -4.41 | 0.000180339 |
| **XLOC_004341** | **Grap** | 11:61653264-61720826 | 0.11 | 2.31 | 4.39 | 0.0225461 |
